# Prevalence of Soil-Transmitted Helminths in Long-Tailed Macaques (*Macaca fascicularis*) in Asia: A Systematic Review and Meta-Analysis

**DOI:** 10.3390/ani16121764

**Published:** 2026-06-08

**Authors:** Issarapong Phosuk, Wipavadee Daiponmak, Darunee Puangpronpitag, Somsiri Potarin, Jurairat Jongthawin

**Affiliations:** 1 Department of Public Health, Amnatcharoen Campus, Mahidol University, Amnat Charoen 37000, Thailand; issarapong.pho@mahidol.ac.th; 2 Faculty of Medicine, Mahasarakham University, Maha Sarakham 44000, Thailand; wipavadee.d@msu.ac.th (W.D.); darunee.p@msu.ac.th (D.P.); 3 International and National Collaborative Network and Innovation for Community Health Development Research Unit, Mahasarakham University, Maha Sarakham 44000, Thailand; 4 Research and Academic Services Group, Amnatcharoen Campus, Mahidol University, Amnat Charoen 37000, Thailand; somsiri.pot@mahidol.ac.th; 5 Biomedical Science Research Unit, Mahasarakham University, Maha Sarakham 44000, Thailand

**Keywords:** *Macaca fascicularis*, soil-transmitted helminths, one health, wildlife, meta-analysis

## Abstract

Long-tailed macaques (*Macaca fascicularis*) commonly inhabit human-modified environments across Asia, where close human–macaque interactions have increased interest in understanding soil-transmitted helminth (STH) infections at human–wildlife interfaces. Because gastrointestinal helminth infections can affect animal health, understanding STH prevalence may support wildlife health surveillance. This systematic review and meta-analysis aimed to estimate the prevalence of major STHs in long-tailed macaques across diverse habitat settings in Asia. EMBASE, PubMed, and Scopus were systematically searched for studies published between 2000 and January 2026, with additional records identified through Google Scholar. Random-effects models with Freeman–Tukey double-arcsine transformation were used to calculate pooled prevalence estimates, and subgroup analyses were performed by habitat type, country, and diagnostic method. Thirty-five studies comprising 5505 macaques from seven countries, predominantly in Southeast Asia, were included. Studies involved urban/temple-associated free-ranging, wild/semi-wild, and captive populations. Among free-ranging macaques, pooled prevalence estimates were 19.3% for *Strongyloides* spp., 12.9% for hookworms, 9.1% for *Trichuris* spp., and 3.3% for *Ascaris* spp. In captive macaque populations, pooled prevalence estimates were 3.6% for *Strongyloides* spp., 2.2% for hookworm, 1.4% for *Ascaris* spp., and 8.2% for *Trichuris* spp. Subgroup analyses by habitat type suggested relatively higher prevalence estimates in urban/temple-associated settings than in wild/semi-wild habitats for several STH taxa. However, substantial between-study heterogeneity (I^2^ > 90% in most analyses), publication bias, and possible small-study effects were identified in some analyses, particularly for hookworm prevalence estimates. These findings indicate that STH infections have been documented among long-tailed macaque populations across diverse habitat settings in Asia. Nevertheless, pooled prevalence estimates should be interpreted cautiously because of substantial heterogeneity, diagnostic variability, and possible publication bias. Future standardized surveillance and molecular investigations may support improved understanding of the prevalence and distribution of STH infections at human–wildlife interfaces.

## Simple Summary

Long-tailed macaques (*Macaca fascicularis*) frequently inhabit urban parks, temple areas, and other human-modified environments across Asia. Close human–macaque interactions in these settings have increased interest in understanding soil-transmitted helminth (STH) infections at human–wildlife interfaces. Because STH infections may affect gastrointestinal health and well-being in macaques, understanding their occurrence may support wildlife health surveillance. This systematic review and meta-analysis evaluated published evidence on major STH infections in long-tailed macaques across different habitat settings, including free-ranging and captive populations. The results showed that STH infections have been documented in both free-ranging and captive populations. Prevalence estimates in captive populations were generally lower for several STH taxa, whereas subgroup analyses according to habitat type suggested relatively higher prevalence in urban/temple-associated settings than in wild/semi-wild habitats. However, substantial heterogeneity among studies, variation in diagnostic methods, and evidence of publication bias indicate that these findings should be interpreted cautiously. Overall, this study highlights the importance of standardized wildlife parasite surveillance and future molecular investigations to improve understanding of the prevalence and distribution of STH infections across human–wildlife interface settings.

## 1. Introduction

Soil-transmitted helminth (STH) infections remain highly prevalent worldwide and contribute substantially to the global burden of neglected tropical diseases (NTDs) [1,2]. In humans, these infections are associated with a range of adverse health outcomes, including chronic gastrointestinal disorders, malnutrition, iron-deficiency anemia, impaired physical growth, and reduced cognitive development, particularly among children in endemic regions [1,2]. The major human-associated STH groups include roundworms (e.g., *Ascaris lumbricoides*), whipworms (e.g., *Trichuris trichiura*), hookworms (e.g., *Ancylostoma duodenale* and *Necator americanus*), and *Strongyloides stercoralis*. These parasites are commonly grouped as STHs because they share similar environmental transmission pathways and overlapping diagnostic and control approaches [1,3]. Although control programs primarily target human populations [4], increasing evidence indicates that wildlife hosts inhabiting shared environments with humans may influence parasite exposure dynamics. Understanding STH epidemiology in wildlife is therefore important within a One Health framework linking human, animal, and environmental health [5].

Long-tailed macaques (*Macaca fascicularis*) are highly adaptable primates widely distributed across Asia and increasingly inhabit human-modified environments, including urban parks, temple complexes, and tourist-associated settings [6,7]. Habitat fragmentation, urban expansion, and food provisioning have intensified human–macaque interactions and may increase exposure to environmentally transmitted parasites, including soil-transmitted helminths (STHs) [6,7,8,9,10,11,12]. STH transmission occurs through infective stages that develop in contaminated soil, and transmission dynamics are influenced by environmental conditions such as climate, sanitation, host density, and environmental disturbance [1,3]. Several STH taxa reported in macaques have also been identified in humans; however, the presence of similar parasites in both hosts does not alone confirm zoonotic transmission or cross-species transmission dynamics [9,10,11,12].

Despite increasing recognition of wildlife in the epidemiology of parasitic infections, reliable estimates of the prevalence and distribution of STHs in long-tailed macaques across different environments remain limited. Previous reviews have primarily provided qualitative overviews of zoonotic pathogens across multiple primate species rather than quantitative, species-specific assessments [13]. Although several gastrointestinal helminths have been reported in long-tailed macaques, the present review focused specifically on four major STH taxa (*Strongyloides* spp., *Trichuris* spp., hookworms, and *Ascaris* spp.), as these parasites were the most consistently reported and provided sufficient data for quantitative synthesis. Other gastrointestinal helminths were reported only sporadically and lacked sufficient epidemiological evidence for robust pooled analyses. Because gastrointestinal helminth infections may affect health and well-being in non-human primates, understanding STH occurrence in long-tailed macaques may support wildlife health surveillance in environments where humans and macaques frequently interact [5,8].

To address these knowledge gaps, we conducted a systematic review and meta-analysis to estimate the prevalence of major STH infections in long-tailed macaques and to examine variation across geographic regions, habitat types, and diagnostic methods. To our knowledge, this is the first systematic review and meta-analysis to quantitatively assess STH prevalence in long-tailed macaques across Asia. By synthesizing currently available evidence, this study provides an overview of the prevalence and distribution of STH infections across habitat settings.

## 2. Materials and Methods

### 2.1. Protocol and Registration

The protocol for this systematic review and meta-analysis was registered in PROSPERO (CRD420251142911). This review was conducted and reported in accordance with the Preferred Reporting Items for Systematic Reviews and Meta-Analyses (PRISMA) guidelines [14].

### 2.2. Review Question

This systematic review aimed to estimate the pooled prevalence of four major STHs—*Trichuris* spp., *Strongyloides* spp., *Ascaris* spp., and hookworm infections—among long-tailed macaques. The review question was formulated according to the Population, Exposure, Comparator, and Outcome (PECO) framework [15]. The population (P) comprised long-tailed macaques, the exposure (E) was STH infection, no comparator (C) was specified, and the outcome (O) was the pooled prevalence of infection reported in the included studies.

### 2.3. Definitions

For the purposes of this review, study settings were categorized into two broad management groups: free-ranging and captive populations. Free-ranging populations were further classified into: (1) urban/temple-associated free-ranging settings, representing human-modified environments characterized by frequent human visitation, food provisioning, and close human–macaque interactions; and (2) wild/semi-wild settings with relatively limited anthropogenic disturbance. Captive populations included macaques housed in zoos, rescue centers, rehabilitation facilities, confiscation centers, and research facilities. Habitat classification was based on descriptions reported in the original studies. In this review, “human–wildlife interfaces” refers to shared environments where humans and long-tailed macaques frequently interact, particularly in urban parks, temple areas, tourist sites, and other human-modified settings [16].

### 2.4. Eligibility Criteria

Studies were eligible for inclusion if they reported prevalence data for at least one of the four targeted soil-transmitted helminths in long-tailed macaques. Only cross-sectional or prevalence studies published from 2000 onward were included. Studies were excluded if they focused on primate species other than long-tailed macaques, involved experimental infection designs, or were reviews, editorials, or conference abstracts without original data. In cases where duplicate datasets were identified across multiple publications, the most comprehensive and recent version was retained. Studies with sample sizes below 20 animals were excluded from the primary meta-analysis to reduce instability associated with extremely small and highly selective datasets, particularly under conditions of substantial between-study heterogeneity. However, additional sensitivity analyses were subsequently performed to evaluate the potential impact of this exclusion criterion on pooled prevalence estimates.

### 2.5. Literature Search and Strategy

A comprehensive literature search was conducted across three electronic databases: PubMed, EMBASE, and Scopus, from database inception to 10 January 2026. The search strategy combined controlled vocabularies (i.e., Medical Subject Headings [MeSH] for PubMed and Embase Subject Headings [Emtree] for EMBASE) with free-text terms using Boolean operators. Database-specific search fields, such as Title/Abstract [tiab] and TITLE-ABS-KEY, were systematically applied to maximize retrieval sensitivity. The core search framework utilized combinations of the following terms: (“*Macaca fascicularis*” OR “long-tailed macaque” OR “cynomolgus macaque” OR macaque*) AND (“soil-transmitted helminth*” OR helminth* OR hookworm* OR *Trichuris* OR *Ascaris* OR *Strongyloides* OR “gastrointestinal parasite*”). Additionally, manual searches were performed via Google Scholar to capture gray literature. No initial language restrictions were imposed. Publication year restrictions were also not applied during the initial database search in order to maximize retrieval sensitivity and minimize potential inconsistencies in database indexing across platforms. Studies published before 2000 were subsequently excluded during the screening stage according to the predefined eligibility criteria. The complete character-by-character search syntax, comprehensive Boolean structures, and exact hit counts for each database are provided in Supplementary Table S1.

### 2.6. Study Selection and Data Extraction

All retrieved records were imported into EndNote version 21.0 (Clarivate Analytics, Philadelphia, PA, USA), and duplicates were removed. The remaining articles were screened based on titles and abstracts, followed by full-text assessment against predefined eligibility criteria. Studies that did not meet the criteria were excluded, with reasons documented. Eligible studies were included in the final review, and relevant data were extracted into Microsoft Excel 2021 (Microsoft Corporation, Redmond, WA, USA) for analysis. For studies reporting data across multiple years, the year with the largest sample size was selected to avoid overlapping data. When studies included multiple habitat types, each habitat was treated as an independent unit for subgroup analysis. Because the included studies variably reported infections as either “hookworm” or genus-level taxa such as *Ancylostoma* spp., the broader term “hookworm” was used throughout this review unless specific taxonomic identification was explicitly reported in the original study. Study selection was performed independently by two reviewers (JJ and IP). Data extraction was conducted by one reviewer (JJ) and independently verified by two reviewers (WD and DP). Any discrepancies were resolved through discussion until consensus was reached.

### 2.7. Risk of Bias Assessment

The methodological quality of included studies was assessed using the Joanna Briggs Institute (JBI) critical appraisal checklist for prevalence studies. Studies with more than 70% “Yes” responses were classified as low risk of bias, those with 50–69% as moderate risk, and those with less than 50% as high risk [17,18].

### 2.8. Data Synthesis

All prevalence estimates included in this meta-analysis were derived from fecal samples collected from long-tailed macaques. Environmental or soil-based contamination studies were not included. Pooled prevalence estimates were calculated using random-effects models with the DerSimonian–Laird method to account for between-study heterogeneity [19]. To avoid pooling biases and account for distinct ecological and veterinary management differences between settings, separate analyses were stratified for free-ranging populations (comprising urban/temple-associated and wild/semi-wild settings) and captive macaque populations. Prior to pooling, prevalence estimates were transformed using the Freeman–Tukey double-arcsine approach to stabilize sampling variances and robustly manage low or zero-event proportions, before being subsequently back-transformed for presentation [20,21].

Statistical heterogeneity was assessed using Cochran’s Q test and quantified using the I^2^ statistic and τ^2^ (between-study variance). I^2^ values of 25%, 50%, and 75% were interpreted as indicating low, moderate, and high heterogeneity, respectively [22]. A *p*-value < 0.10 for Cochran’s Q test was considered indicative of statistically significant heterogeneity. Subgroup analyses were performed descriptively to estimate pooled prevalence according to predefined study characteristics, including country, habitat type, and diagnostic method for each STH group. Habitat subgroup analyses were restricted to free-ranging macaque populations and categorized as urban/temple-associated free-ranging settings or wild/semi-wild settings. Univariable mixed-effects meta-regression models were further performed separately for each moderator variable (country group, diagnostic method, and habitat type) using restricted maximum likelihood (REML) estimation to evaluate their potential contribution to between-study heterogeneity. Sensitivity analyses were conducted using a leave-one-out approach, in which individual studies were sequentially removed to evaluate the robustness and stability of pooled prevalence estimates. Additional sensitivity analyses were also performed by re-including excluded small-sample studies to assess the potential influence of exclusion criteria on the overall findings. Publication bias was evaluated through visual inspection of funnel plots and Egger’s regression asymmetry test [23]. Where funnel plot asymmetry suggested potential publication bias, the trim-and-fill method was applied to estimate the number of potentially missing studies and to assess their impact on pooled prevalence estimates [24]. All statistical analyses were conducted using RStudio (Version 2024.04.2+764) [25].

## 3. Results

### 3.1. Search Results

**Figure 1:**
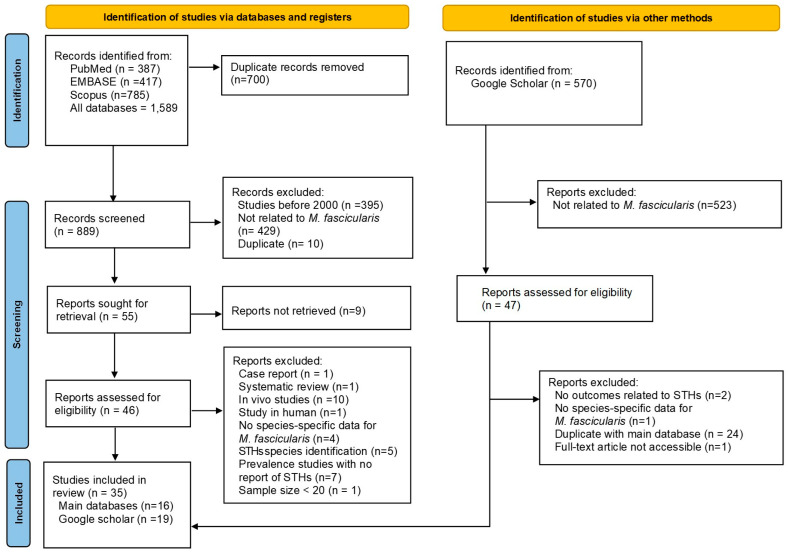
The PRISMA 2020 flow diagram [14] outlines the process of study selection for the review.

A total of 1589 records were identified through searches of EMBASE, PubMed, and Scopus. After removal of duplicates, 889 records remained for title and abstract screening. Following screening and eligibility assessment, 16 studies from the primary database search met the inclusion criteria. In addition, 570 records were identified through Google Scholar. After removal of irrelevant records, 47 remained for eligibility assessment, of which 19 studies met the inclusion criteria. Overall, 35 studies were included in the final systematic review and meta-analysis [6,7,26,27,28,29,30,31,32,33,34,35,36,37,38,39,40,41,42,43,44,45,46,47,48,49,50,51,52,53,54,55,56,57,58]. Detailed exclusion categories and study selection processes are summarized in the PRISMA flow diagram (Figure 1).

### 3.2. Characteristics of Included Studies

All included studies employed cross-sectional designs and were published between 2006 and 2026. Most studies originated from Southeast Asia, particularly Indonesia and Thailand. Free-ranging populations, especially urban/temple-associated settings, were more frequently studied than captive or wild/semi-wild populations. One study contributed data from multiple habitat categories [26]. Regarding diagnostic approaches, sedimentation-based fecal concentration techniques were the most commonly reported methods, followed by flotation techniques and combined diagnostic approaches. Direct smear microscopy was used less frequently (Table 1).

### 3.3. Risk of Bias Assessment Results

Most of the included studies (29/35; 82.9%) showed a low risk of bias, while 6 studies (17.1%) had a moderate risk of bias (Table S3). No study was assessed as having a high risk of bias. Therefore, all studies were included in the systematic review and meta-analysis.

### 3.4. Pooled Prevalence of Soil-Transmitted Helminth Infections in Long-Tailed Macaques

**Figure 2:**
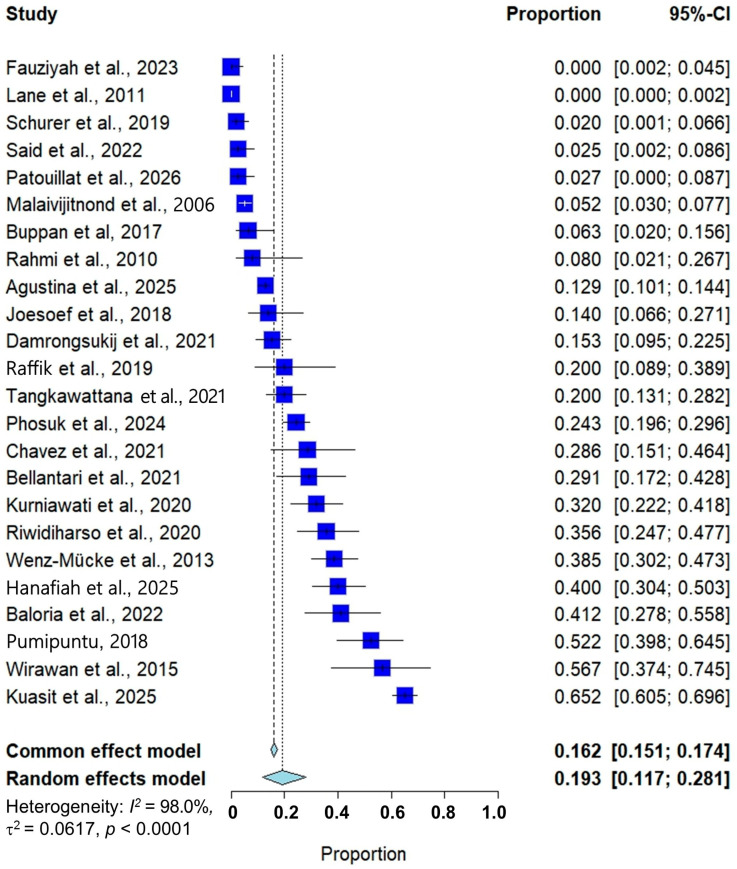
Forest plot of the prevalence of *Strongyloides* spp. infections in free-ranging long-tailed macaques across 24 studies in Southeast Asia. Each horizontal line represents the 95% confidence interval (CI) of an individual study, with blue squares indicating point estimates. The pooled prevalence, estimated using both fixed- (common) and random-effects models, is represented by the diamond [6,7,26,27,28,29,30,31,32,33,34,35,36,37,38,39,40,41,42,43,44,45,46,47].

**Figure 3:**
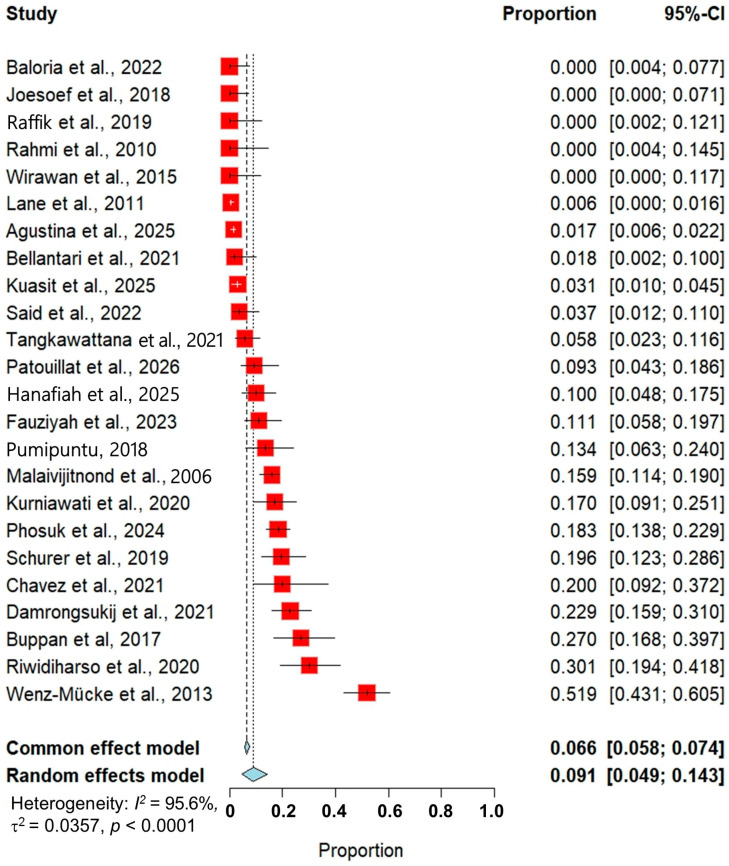
Forest plot of the prevalence of *Trichuris* spp. infections in free-ranging long-tailed macaques across 24 studies in Southeast Asia. Each horizontal line represents the 95% confidence interval (CI) of an individual study, with red squares indicating the point estimates. The pooled prevalence is shown as a diamond, estimated using both fixed- (common) and random-effects models [6,7,26,27,28,29,30,31,32,33,34,35,36,37,38,39,40,41,42,43,44,45,46,47].

**Figure 4:**
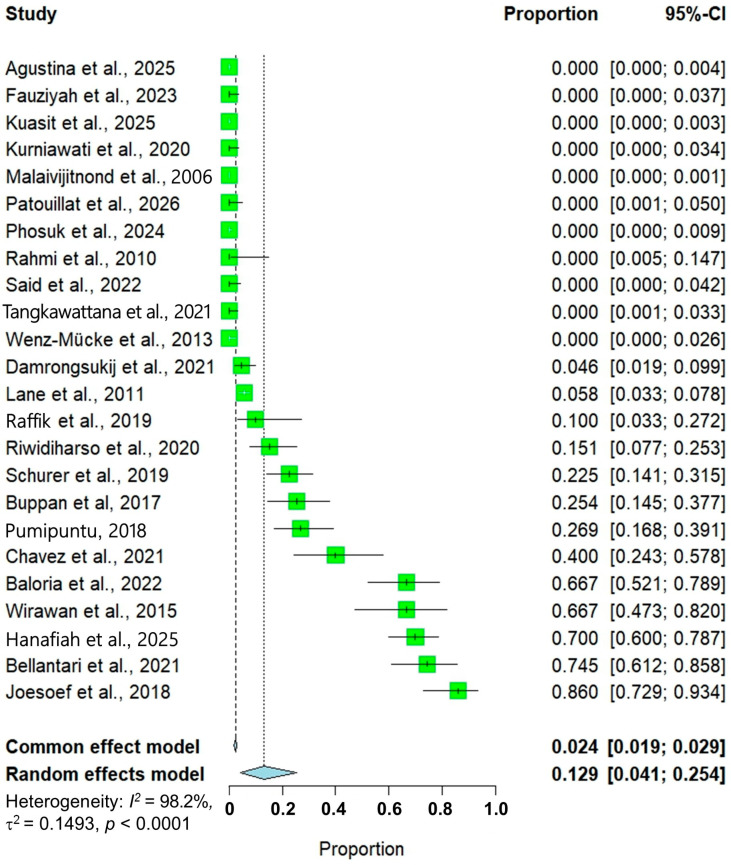
Forest plot of the prevalence of hookworm infections in free-ranging long-tailed macaques across 24 studies in Southeast Asia. Each horizontal line represents the 95% confidence interval (CI) of an individual study, with green squares indicating the point estimates. The pooled prevalence is shown as a diamond, estimated using both fixed- (common) and random-effects models [6,7,26,27,28,29,30,31,32,33,34,35,36,37,38,39,40,41,42,43,44,45,46,47].

**Figure 5:**
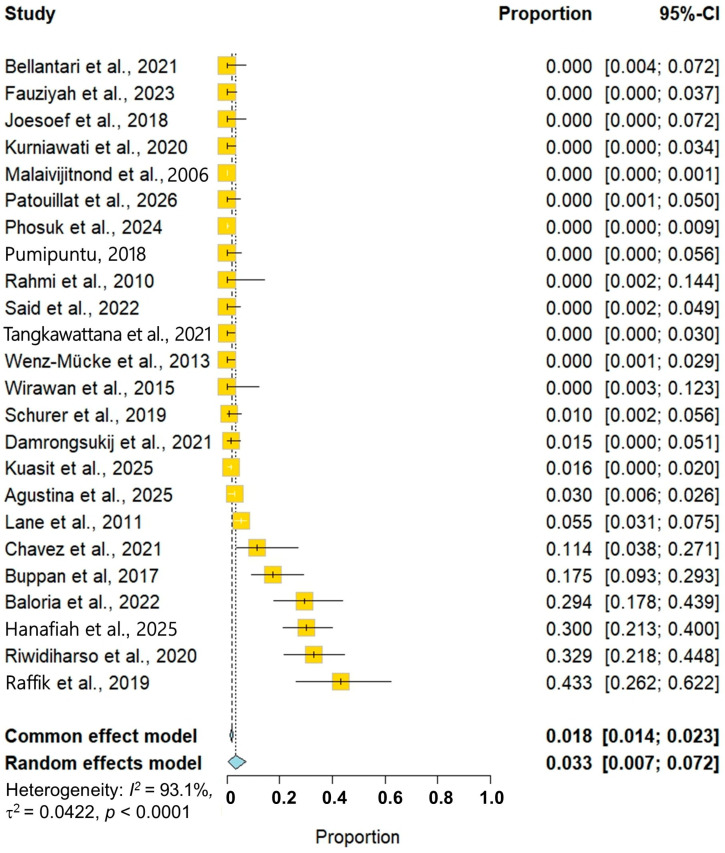
Forest plot of the prevalence of *Ascaris* spp. infections in free-ranging long-tailed macaques across 24 studies in Southeast Asia. Each horizontal line represents the 95% confidence interval (CI) of an individual study, with gold squares indicating the point estimates. The pooled prevalence is shown as a diamond, estimated using both fixed- (common) and random-effects models [6,7,26,27,28,29,30,31,32,33,34,35,36,37,38,39,40,41,42,43,44,45,46,47].

A total of 5505 long-tailed macaques from both free-ranging settings (urban/temple-associated free-ranging settings, *n* = 3408; wild/semi-wild free-ranging settings, *n* = 631) [6,7,26,27,28,29,30,31,32,33,34,35,36,37,38,39,40,41,42,43,44,45,46,47] and captive settings (*n* = 1466) [48,49,50,51,52,53,54,55,56,57,58] were included in this meta-analysis. Because substantial heterogeneity was observed across studies (I^2^ > 90% in most analyses), pooled prevalence estimates were calculated using random-effects models. Among free-ranging macaque populations, the pooled prevalence estimates were 19.3% for *Strongyloides* spp., 9.1% for *Trichuris* spp., 12.9% for hookworm, and 3.3% for *Ascaris* spp. (Figure 2, Figure 3, Figure 4 and Figure 5). In captive macaque populations, pooled prevalence estimates were 3.6% for *Strongyloides* spp., 2.2% for hookworm, 1.4% for *Ascaris* spp., and 8.2% for *Trichuris* spp. (Supplementary File S1).

### 3.5. Subgroup Analysis of STH Infections in Free-Ranging Long-Tailed Macaques by Country, Habitat and Diagnostic Method

Subgroup analyses were performed by country, habitat type, and diagnostic method among free-ranging long-tailed macaque populations. Significant between-subgroup heterogeneity was observed across subgroup analyses (*p* < 0.001). Country-level subgroup analyses suggested variation in prevalence estimates across study settings, with relatively higher prevalence estimates for *Strongyloides* spp., hookworms, and *Ascaris* spp. reported in studies from the Philippines, whereas studies from Thailand generally reported relatively higher *Trichuris* spp. prevalence estimates. However, these findings should be interpreted cautiously because several country subgroups were represented by limited numbers of studies. Habitat subgroup analyses suggested relatively higher prevalence estimates for *Strongyloides* spp., *Trichuris* spp., and hookworms in urban/temple-associated settings than in wild/semi-wild habitats, whereas *Ascaris* spp. showed relatively higher prevalence estimates in wild/semi-wild settings. Differences in prevalence estimates were also observed across diagnostic-method categories. Direct smear and sedimentation methods generally yielded higher prevalence estimates for *Strongyloides* spp., *Trichuris* spp., and hookworms than flotation methods, although these differences may partially reflect variation in diagnostic sensitivity rather than true ecological variation. Detailed pooled prevalence estimates and 95% confidence intervals for all subgroup analyses are presented in Table 2 and Supplementary File S2.

### 3.6. Meta-Regression Analysis of Factors Associated with Prevalence Heterogeneity

Meta-regression analyses were conducted to explore potential sources of between-study heterogeneity, including country group, diagnostic method, and habitat type. Substantial residual heterogeneity persisted across all models (I^2^ > 90%). No significant associations were observed for *Strongyloides* spp., hookworms, or *Trichuris* spp. prevalence (*p* > 0.05), although studies from Thailand tended to report relatively higher *Trichuris* spp. estimates. For *Ascaris* spp., country group significantly contributed to heterogeneity (*p* = 0.0294), while diagnostic method and habitat type were not significant moderators. Overall, heterogeneity remained largely unexplained across analyses (Supplementary File S3).

### 3.7. Sensitivity Analysis

Leave-one-out sensitivity analyses showed that pooled prevalence estimates remained broadly stable across all parasite groups in both free-ranging (Tables S4–S7) and captive macaque populations (Tables S8–S11), with no single study substantially influencing the overall results. However, substantial between-study heterogeneity (I^2^ > 95%) persisted throughout the analyses. To evaluate the potential impact of excluding studies with sample sizes below 20 animals, an additional sensitivity analysis was performed by re-including the excluded study by Pumipuntu et al. (2013) (*n* = 11) [59]. Following re-analysis, the pooled prevalence estimates for *Strongyloides* spp. decreased slightly from 19.3% to 18.4%, whereas prevalence estimates for the remaining STH taxa remained largely unchanged. These findings suggest that exclusion of the small-sample study had minimal influence on the overall results (Supplementary File S4).

### 3.8. Publication Bias Analysis

Publication bias was assessed for each STH taxon using Egger’s regression test and visual inspection of funnel plots. In free-ranging long-tailed macaque populations, evidence of funnel plot asymmetry was identified for hookworm prevalence estimates (*p* = 0.0006). Trim-and-fill analysis reduced the adjusted pooled prevalence estimate from 12.9% to 1.0% (95% CI: 0.0–10.3%), suggesting possible publication bias, small-study effects, or instability associated with substantial between-study heterogeneity. No statistically significant evidence of publication bias was detected for *Strongyloides* spp., *Trichuris* spp., or *Ascaris* spp. (Supplementary File S5). In captive long-tailed macaque populations, evidence of funnel plot asymmetry was identified for *Trichuris* spp. prevalence estimates (*p* = 0.0161). Trim-and-fill analysis reduced the adjusted pooled prevalence estimate from 8.2% to 2.3% (95% CI: 0.0–7.7%). No statistically significant evidence of publication bias was detected for *Strongyloides* spp., hookworms, or *Ascaris* spp. (Supplementary File S6).

## 4. Discussion

This systematic review and meta-analysis provides the first quantitative synthesis of major STH infections in long-tailed macaques across Asia. A total of 5505 macaques from both free-ranging and captive settings were included. Overall, STH infections were documented across diverse habitat settings, although prevalence patterns differed between management conditions. Among free-ranging macaques, *Strongyloides* spp. showed the highest pooled prevalence, whereas prevalence estimates in captive populations were generally lower for several STH taxa. These differences may partially reflect environmental conditions, enclosure sanitation, veterinary management, and possible routine antiparasitic treatment in some facilities. However, because information regarding deworming practices and treatment history was inconsistently reported across studies, these explanations remain tentative. Moreover, substantial between-study heterogeneity persisted across analyses and should be considered when interpreting pooled prevalence estimates.

Evidence of publication bias was observed for hookworm prevalence estimates in free-ranging macaques and *Trichuris* spp. prevalence estimates in captive populations. Trim-and-fill analyses reduced adjusted pooled prevalence estimates, suggesting possible overrepresentation of studies reporting higher infection levels. This asymmetry may reflect selective reporting, methodological variability, sparse-event data, or instability associated with substantial between-study heterogeneity [24]. Therefore, these prevalence estimates should be interpreted as broad indicators of reported infection patterns rather than precise estimates of underlying infection burden.

Subgroup analyses according to country, habitat type, and diagnostic method suggested variation in prevalence estimates across study settings. Country-level differences were observed across several STH taxa; however, these findings should be interpreted cautiously because the number of available studies varied substantially among countries and several subgroups were represented by limited numbers of studies, restricting meaningful geographic comparisons. Meta-regression analyses further indicated that country group significantly contributed to heterogeneity only for *Ascaris* spp., whereas no significant associations were identified for other STH taxa. Overall, substantial unexplained heterogeneity remained across models.

Subgroup analyses also suggested relatively higher prevalence estimates for several STH taxa in urban/temple-associated settings than in wild/semi-wild habitats. Human-modified environments characterized by food provisioning, high macaque population density, and frequent human visitation may increase opportunities for environmental contamination and repeated exposure to infective stages. However, habitat type was not identified as a significant independent moderator in meta-regression analyses, suggesting that ecological, behavioral, and methodological factors likely acted simultaneously to influence prevalence patterns across studies. Therefore, these findings should be interpreted cautiously and not as evidence of direct environmental causality.

Variation in diagnostic methods likely contributed substantially to the observed heterogeneity. Most included studies relied on conventional microscopy-based techniques, including sedimentation, flotation, direct smear, or combinations of these approaches, which differ in analytical sensitivity and species-level resolution [60,61]. In addition, variability in microscopy-based identification and challenges in differentiating larval-stage nematodes, particularly *Strongyloides* spp. and hookworms, may have influenced taxonomic identification and prevalence estimates across studies [62]. Consequently, differences observed across diagnostic-method subgroups may partially reflect methodological variation rather than true differences in underlying prevalence patterns. Diagnostic method was not identified as a significant independent moderator in meta-regression analyses, although interpretation may have been limited by small subgroup sizes and residual between-study variability. These findings support the need for standardized diagnostic protocols and greater incorporation of molecular approaches in future wildlife parasitology studies [62].

From a broader One Health perspective, many included studies were conducted in environments characterized by close human–macaque interactions [6,7,26,27,28,29,31,34,35,37,38,39,40,43,44,45,46,47]. Future surveillance efforts may benefit from standardized fecal sampling, integration of molecular approaches, environmental monitoring, and coordinated collaboration across wildlife, veterinary, environmental, and public health sectors [5]. Such approaches may improve understanding of parasite exposure patterns at human–wildlife interfaces and support more integrated wildlife health surveillance.

Several limitations should be considered when interpreting these findings. First, substantial between-study heterogeneity remained across most analyses despite subgroup and meta-regression approaches, indicating that important ecological and methodological sources of variation were not fully explained. Second, most included studies relied primarily on microscopy-based diagnostic methods, which have variable analytical sensitivity and limited species-level resolution. In particular, overlapping microscopic features and limitations of routine fecal examination for some STH taxa, especially *Strongyloides* spp. and hookworms, may have affected taxonomic classification and prevalence estimates across studies. Third, many included studies were designed as general gastrointestinal parasite surveys rather than investigations specifically targeting individual STH taxa, which may have introduced inconsistency in sampling approaches and reporting practices. Fourth, geographic representation was uneven, with most studies originating from a limited number of Southeast Asian countries, potentially limiting broader regional generalizability. Finally, publication bias and possible small-study effects were identified for some STH taxa and may have influenced pooled prevalence estimates.

## 5. Conclusions

This study presents the first systematic review and meta-analysis of major STH infections in long-tailed macaques across Asia. *Strongyloides* spp., *Trichuris* spp., hookworms, and *Ascaris* spp. were widely reported across diverse habitat settings, with subgroup analyses suggesting relatively higher prevalence estimates in urban/temple-associated settings than in wild/semi-wild habitats. However, these findings should be interpreted cautiously because substantial heterogeneity, methodological variability, and evidence of publication bias were identified across analyses. Overall, these findings provide baseline epidemiological data supporting the need for standardized surveillance and molecular investigations of STH infections in long-tailed macaques.

## Figures and Tables

**Table 1:** Summary Characteristics of Included Studies (*n* = 35).

Characteristic	*n*	%
**Publication Year**		
2006–2019	18	51.4
2020–2026	17	48.6
**Countries**		
Indonesia	17	48.6
Thailand	9	25.7
Philippines	3	8.6
Malaysia	2	5.7
China	2	5.7
Republic of Korea	1	2.9
Russia	1	2.9
**Habitats**		
Urban/temple-associated free-ranging settings	18 *	51.4
Wild/semi-wild settings	7 *	20.0
Captive settings	11	31.4
**Diagnostic methods**		
Sedimentation methods (alone or with other methods)	17	48.6
Flotation methods (alone or with other methods)	10	28.6
Combination (sedimentation and flotation with other methods)	5	14.3
Direct smear	3	8.6

* One study reported data from both urban/temple-associated and wild/semi-wild settings [26].

**Table 2:** Subgroup Analysis of STH Infections in Free-Ranging Long-Tailed Macaques by Country, Habitat and Diagnostic Method.

Parasite	Category	Subgroup	Studies (n)	Pooled Prevalence (%)	95% CI	I^2^
*Strongyloides* spp.	Country	Indonesia	11	16.3	6.1, 29.9	97.3
		Philippines	2	35.6	24.0, 48.1	27.4
		Thailand	9	22.5	9.7, 38.6	98.6
		Malaysia	2	8.8	0.0, 32.4	87.4
	Habitat *	Urban/temple	18	19.4	10.5, 30.1	98.4
		Wild/semi-wild	7	18.7	7.5, 33.2	93.5
	Diagnostic method	Sedimentation	15	22.7	13.6, 33.4	97.9
		Flotation	5	7.4	0.1, 22.5	96.8
		Combination	2	8.8	0.0, 53.7	97.3
		Direct smear	2	43.7	28.0, 60.2	74.2
*Trichuris* spp.	Country	Indonesia	11	5.2	1.4, 10.9	92.2
		Philippines	2	6.1	0.0, 37.8	92.7
		Thailand	9	18.0	9.8, 28.0	96.0
		Malaysia	2	1.9	0.0, 6.7	19.6
	Habitat *	Urban/temple	18	9.4	4.4, 16.1	96.4
		Wild/semi-wild	7	6.4	1.4, 13.9	88.9
	Diagnostic method	Sedimentation	15	9.9	4.1, 17.6	96.5
		Flotation	5	4.5	0.4, 12.0	91.7
		Combination	2	5.9	0.1, 17.6	78.6
		Direct smear	2	21.3	7.4, 39.5	82.4
Hookworm	Country	Indonesia	11	19.2	2.5, 45.3	98.8
		Philippines	2	53.9	28.1, 78.7	83.0
		Thailand	9	4.2	0.1, 12.7	96.1
		Malaysia	2	2.6	0.0, 19.8	86.2
	Habitat *	Urban/temple	18	13.2	3.1, 28.2	98.1
		Wild/semi-wild	7	9.2	0.0, 31.9	98.2
	Diagnostic method	Sedimentation	15	15.9	3.3, 34.9	98.6
		Flotation	5	1.4	0.0, 5.6	85.3
		Combination	2	26.4	0.0, 100.0	99.3
		Direct smear	2	20.6	10.3, 33.2	65.5
*Ascaris* spp.	Country	Indonesia	11	3.0	0.0, 9.2	93.2
		Philippines	2	20.2	5.7, 39.9	74.6
		Thailand	9	0.8	0.0, 3.0	83.5
		Malaysia	2	13.3	0.0, 74.6	97.4
	Habitat *	Urban/temple	18	2.1	0.2, 5.4	91.6
		Wild/semi-wild	7	7.5	0.2, 21.6	94.4
	Diagnostic method	Sedimentation	15	3.0	0.3, 7.6	93.0
		Flotation	5	4.1	0.0, 19.3	93.1
		Combination	2	0	0.0, 1.3	0
		Direct smear	2	10.4	0.0, 59.4	97.7

*p*-value between subgroups for all category < 0.001, * One study reported data from both urban/temple-associated and wild/semi-wild settings [26].

## Data Availability

The data underlying the results of this study are publicly available within the article and its Supplementary Materials.

## Supplementary Materials

The following supporting information can be downloaded at: https://www.mdpi.com/article/10.3390/ani16121764/s1, Table S1: Search strategies used for each database. Table S2: Details of the included studies. Table S3: Methodological quality assessment of included studies. Tables S4–S7: Leave-one-out analysis of STHs in free-ranging long-tail macaque. Tables S8–S11: Leave-one-out meta-analysis of STHs in captive long-tail macaques. Supplementary File S1: Pooled prevalence of STHs in captive long-tailed macaque. Supplementary File S2: Subgroup analysis of STHs. Supplementary File S3: Meta-regression. Supplementary File S4: Sensitivity analysis including the excluded small-sample study for STHs prevalence. Supplementary File S5: Funnel plots of STHs prevalence in free-ranging long-tailed macaque. Supplementary File S6: Funnel plots of STHs prevalence in captive.
