# Supplementary material for: Prevalence of Soil-Transmitted Helminths in Long-Tailed Macaques (Macaca fascicularis) in Asia: A Systematic Review and Meta-Analysis
Source: Animals (Basel). 2026 Jun 8;16(12):1764. doi: 10.3390/ani16121764 (PMC13295248; doi:10.3390/ani16121764)
Supplement: Supplementary file 1 [file animals-16-01764-s001.zip › Table S4-S7. Leave-one-out analysis of STHs in free-ranging long-tail macaque_edited2.pdf]

Table S4. Leave-one-out meta-analysis of *Strongyloides* spp.in free-ranging long-tail macaque

	proportion	95%-CI	tau^2
Omitting Agustina et al., 2025	0.1958	[0.1169; 0.2885]	0.0643
Omitting Baloria et al., 2022	0.1845	[0.1091; 0.2736]	0.0620
Omitting Bellantari et al., 2021	0.1887	[0.1115; 0.2799]	0.0639
Omitting Buppan et al, 2017	0.1993	[0.1207; 0.2912]	0.0629
Omitting Chavez et al., 2021	0.1891	[0.1120; 0.2802]	0.0639
Omitting Damrongsukij et al., 2021	0.1945	[0.1158; 0.2870]	0.0645
Omitting Fauziyah et al., 2023	0.2070	[0.1304; 0.2953]	0.0568
Omitting Hanafiah et al., 2025	0.1845	[0.1089; 0.2739]	0.0622
Omitting Joesoef et al., 2018	0.1949	[0.1164; 0.2872]	0.0643
Omitting Kuasit et al., 2025	0.1747	[0.1059; 0.2558]	0.0528
Omitting Kurniawati et al., 2020	0.1874	[0.1106; 0.2783]	0.0636
Omitting Malaivijitnond et al., 2006	0.2008	[0.1222; 0.2924]	0.0621
Omitting Patouillat et al., 2026	0.2024	[0.1242; 0.2933]	0.0610
Omitting Phosuk et al., 2024	0.1904	[0.1124; 0.2824]	0.0645
Omitting Pumipuntu , 2018	0.1805	[0.1074; 0.2669]	0.0590
Omitting Raffik et al., 2019	0.1923	[0.1144; 0.2841]	0.0644
Omitting Rahmi et al., 2010	0.1976	[0.1192; 0.2894]	0.0635
Omitting Riwidiharso et al., 2020	0.1862	[0.1099; 0.2764]	0.0630
Omitting Said et al., 2022	0.2026	[0.1245; 0.2934]	0.0609
Omitting Tangkawattana et.al 2021	0.1923	[0.1139; 0.2845]	0.0646
Omitting Schurer et al., 2019	0.2034	[0.1255; 0.2939]	0.0603
Omitting Wenz-Mücke et al., 2013	0.1849	[0.1091; 0.2746]	0.0625
Omitting Wirawan et al., 2015	0.1802	[0.1077; 0.2658]	0.0583
Omitting Lane et al., 2011	0.2086	[0.1327; 0.2958]	0.0550
Random effects model	0.1925	[0.1172; 0.2806]	0.0617

Table S5. Leave-one-out meta-analysis of *Trichuris* spp. in free-ranging long-tail macaque

	proportion	95%-CI	tau^2
Omitting Agustina et al., 2025	0.0956	[0.0517; 0.1502]	0.0357
Omitting Baloria et al., 2022	0.0969	[0.0533; 0.1507]	0.0347
Omitting Bellantari et al., 2021	0.0945	[0.0507; 0.1491]	0.0364
Omitting Buppan et al, 2017	0.0847	[0.0438; 0.1363]	0.0350
Omitting Chavez et al., 2021	0.0871	[0.0451; 0.1400]	0.0364
Omitting Damrongsukij et al., 2021	0.0855	[0.0439; 0.1379]	0.0359
Omitting Fauziyah et al., 2023	0.0896	[0.0463; 0.1441]	0.0375
Omitting Hanafiah et al., 2025	0.0901	[0.0466; 0.1447]	0.0375
Omitting Joesoef et al., 2018	0.0969	[0.0533; 0.1507]	0.0348
Omitting Kuasit et al., 2025	0.0941	[0.0501; 0.1490]	0.0366
Omitting Kurniawati et al., 2020	0.0874	[0.0449; 0.1411]	0.0369
Omitting Malaivijitnond et al., 2006	0.0876	[0.0449; 0.1416]	0.0372
Omitting Patouillat et al., 2026	0.0904	[0.0469; 0.1450]	0.0375
Omitting Phosuk et al., 2024	0.0868	[0.0444; 0.1403]	0.0368
Omitting Pumipuntu , 2018	0.0888	[0.0458; 0.1429]	0.0373
Omitting Raffik et al., 2019	0.0961	[0.0525; 0.1501]	0.0354
Omitting Rahmi et al., 2010	0.0958	[0.0523; 0.1499]	0.0356
Omitting Riwidiharso et al., 2020	0.0838	[0.0435; 0.1346]	0.0342
Omitting Said et al., 2022	0.0933	[0.0494; 0.1481]	0.0369
Omitting Tangkawattana et.al 2021	0.0921	[0.0483; 0.1471]	0.0373
Omitting Schurer et al., 2019	0.0866	[0.0445; 0.1397]	0.0365
Omitting Wenz-Mücke et al., 2013	0.0781	[0.0435; 0.1209]	0.0253
Omitting Wirawan et al., 2015	0.0961	[0.0525; 0.1501]	0.0354
Omitting Lane et al., 2011	0.0970	[0.0532; 0.1510]	0.0346
Random effects model	0.0906	[0.0487; 0.1427]	0.0357

Table S6. Leave-one-out meta-analysis of Hookworm in free-ranging long-tail macaque

	proportion	95%-CI	tau^2
Omitting Agustina et al., 2025	0.1405	[0.0463; 0.2715]	0.1500
Omitting Baloria et al., 2022	0.1128	[0.0317; 0.2309]	0.1405
Omitting Bellantari et al., 2021	0.1102	[0.0312; 0.2253]	0.1354
Omitting Buppan et al, 2017	0.1249	[0.0358; 0.2536]	0.1550
Omitting Chavez et al., 2021	0.1207	[0.0341; 0.2463]	0.1516
Omitting Damrongsukij et al., 2021	0.1341	[0.0412; 0.2656]	0.1552
Omitting Fauziyah et al., 2023	0.1394	[0.0453; 0.2705]	0.1513
Omitting Hanafiah et al., 2025	0.1112	[0.0312; 0.2279]	0.1379
Omitting Joesoef et al., 2018	0.1063	[0.0309; 0.2156]	0.1259
Omitting Kuasit et al., 2025	0.1403	[0.0461; 0.2713]	0.1503
Omitting Kurniawati et al., 2020	0.1394	[0.0454; 0.2706]	0.1513
Omitting Malaivijitnond et al., 2006	0.1402	[0.0460; 0.2713]	0.1504
Omitting Patouillat et al., 2026	0.1392	[0.0452; 0.2703]	0.1515
Omitting Phosuk et al., 2024	0.1401	[0.0459; 0.2712]	0.1505
Omitting Pumipuntu , 2018	0.1244	[0.0355; 0.2528]	0.1548
Omitting Raffik et al., 2019	0.1308	[0.0393; 0.2614]	0.1560
Omitting Rahmi et al., 2010	0.1380	[0.0445; 0.2690]	0.1527
Omitting Riwidiharso et al., 2020	0.1287	[0.0377; 0.2590]	0.1562
Omitting Said et al., 2022	0.1393	[0.0453; 0.2704]	0.1515
Omitting Tangkawattana et.al 2021	0.1396	[0.0455; 0.2707]	0.1511
Omitting Schurer et al., 2019	0.1258	[0.0361; 0.2551]	0.1555
Omitting Wenz-Mücke et al., 2013	0.1397	[0.0456; 0.2708]	0.1510
Omitting Wirawan et al., 2015	0.1134	[0.0321; 0.2319]	0.1412
Omitting Lane et al., 2011	0.1334	[0.0406; 0.2650]	0.1556
Random effects model	0.1295	[0.0412; 0.2539]	0.1493

Table S7. Leave-one-out meta-analysis of *Ascaris* spp. in free-ranging long-tail macaque

	proportion	95%-CI	tau^2
Omitting Agustina et al., 2025	0.0328	[0.0059; 0.0752]	0.0445
Omitting Baloria et al., 2022	0.0266	[0.0042; 0.0625]	0.0375
Omitting Bellantari et al., 2021	0.0348	[0.0073; 0.0773]	0.0436
Omitting Buppan et al, 2017	0.0286	[0.0045; 0.0675]	0.0415
Omitting Chavez et al., 2021	0.0303	[0.0052; 0.0706]	0.0431
Omitting Damrongsukij et al., 2021	0.0335	[0.0064; 0.0760]	0.0443
Omitting Fauziyah et al., 2023	0.0350	[0.0074; 0.0776]	0.0434
Omitting Hanafiah et al., 2025	0.0259	[0.0040; 0.0612]	0.0366
Omitting Joesoef et al., 2018	0.0348	[0.0073; 0.0773]	0.0436
Omitting Kuasit et al., 2025	0.0336	[0.0064; 0.0762]	0.0443
Omitting Kurniawati et al., 2020	0.0351	[0.0075; 0.0777]	0.0434
Omitting Malaivijitnond et al., 2006	0.0355	[0.0077; 0.0781]	0.0429
Omitting Patouillat et al., 2026	0.0350	[0.0074; 0.0775]	0.0435
Omitting Phosuk et al., 2024	0.0354	[0.0077; 0.0781]	0.0430
Omitting Pumipuntu , 2018	0.0349	[0.0074; 0.0775]	0.0435
Omitting Raffik et al., 2019	0.0252	[0.0044; 0.0582]	0.0334
Omitting Rahmi et al., 2010	0.0345	[0.0073; 0.0767]	0.0438
Omitting Riwidiharso et al., 2020	0.0256	[0.0041; 0.0602]	0.0356
Omitting Said et al., 2022	0.0350	[0.0074; 0.0776]	0.0434
Omitting Tangkawattana et.al 2021	0.0351	[0.0075; 0.0778]	0.0433
Omitting Schurer et al., 2019	0.0339	[0.0066; 0.0764]	0.0442
Omitting Wenz-Mücke et al., 2013	0.0352	[0.0075; 0.0778]	0.0432
Omitting Wirawan et al., 2015	0.0346	[0.0073; 0.0769]	0.0438
Omitting Lane et al., 2011	0.0316	[0.0054; 0.0735]	0.0444
Random effects model	0.0325	[0.0067; 0.0724]	0.0422
