# Supplementary material for: Prevalence of Soil-Transmitted Helminths in Long-Tailed Macaques (Macaca fascicularis) in Asia: A Systematic Review and Meta-Analysis
Source: Animals (Basel). 2026 Jun 8;16(12):1764. doi: 10.3390/ani16121764 (PMC13295248; doi:10.3390/ani16121764)
Supplement: Supplementary file 1 [file animals-16-01764-s001.zip › Table S8-S11. Leave-one-out meta-analysis of STHs in captive long tail macaques.pdf]

Table S8. Leave-one-out meta-analysis of *Strongyloides* spp.in captive settings

	proportion	95%-CI	tau^2
Omitting Casim et al., 2015	0.0323	[0.0000; 0.1212]	0.0585
Omitting Dwipayanti et al., 2014	0.0425	[0.0000; 0.1361]	0.0567
Omitting Ferdinandez et al., 2023	0.0419	[0.0000; 0.1351]	0.0572
Omitting Kumalasari et al., 2010	0.0311	[0.0000; 0.1172]	0.0569
Omitting lee et al., 2010	0.0418	[0.0000; 0.1349]	0.0573
Omitting Li et al., 2015	0.0331	[0.0000; 0.1215]	0.0582
Omitting Purnama et al., 2021	0.0417	[0.0000; 0.1347]	0.0574
Omitting Rosyid et al., 2023	0.0416	[0.0000; 0.1344]	0.0574
Omitting Teo et al., 2019	0.0110	[0.0000; 0.0362]	0.0078
Omitting Zanzani et al., 2016	0.0449	[0.0000; 0.1383]	0.0538
Omitting Egorova 2010	0.0389	[0.0000; 0.1337]	0.0593
Random effects model	0.0358	[0.0000; 0.1162]	0.0520

Table S9. Leave-one-out meta-analysis of *Trichuris* spp. in captive settings

	proportion	95%-CI	tau^2
Omitting Casim et al., 2015	0.0746	[0.0289; 0.1356]	0.0163
Omitting Dwipayanti et al., 2014	0.0689	[0.0276; 0.1235]	0.0132
Omitting Ferdinandez et al., 2023	0.0808	[0.0324; 0.1449]	0.0177
Omitting Kumalasari et al., 2010	0.0932	[0.0445; 0.1550]	0.0145
Omitting lee et al., 2010	0.0834	[0.0342; 0.1483]	0.0178
Omitting Li et al., 2015	0.0868	[0.0370; 0.1519]	0.0174
Omitting Purnama et al., 2021	0.0767	[0.0307; 0.1377]	0.0165
Omitting Rosyid et al., 2023	0.0731	[0.0297; 0.1306]	0.0149
Omitting Teo et al., 2019	0.0777	[0.0317; 0.1386]	0.0166
Omitting Zanzani et al., 2016	0.0954	[0.0469; 0.1562]	0.0124
Omitting Egorova 2010	0.0906	[0.0393; 0.1572]	0.0164
Random effects model	0.0817	[0.0363; 0.1403]	0.0158

Table S10. Leave-one-out meta-analysis of Hookworm in captive settings

	proportion	95%-CI	tau^2
Omitting Casim et al., 2015	0.0263	[0.0000; 0.1656]	0.1349
Omitting Dwipayanti et al., 2014	0.0000	[0.0000; 0.0000]	0
Omitting Ferdinandez et al., 2023	0.0259	[0.0000; 0.1642]	0.1352
Omitting Kumalasari et al., 2010	0.0259	[0.0000; 0.1641]	0.1352
Omitting lee et al., 2010	0.0259	[0.0000; 0.1640]	0.1352
Omitting Li et al., 2015	0.0259	[0.0000; 0.1638]	0.1352
Omitting Purnama et al., 2021	0.0259	[0.0000; 0.1637]	0.1352
Omitting Rosyid et al., 2023	0.0258	[0.0000; 0.1634]	0.1353
Omitting Teo et al., 2019	0.0257	[0.0000; 0.1628]	0.1353
Omitting Zanzani et al., 2016	0.0277	[0.0000; 0.1686]	0.1335
Omitting Egorova 2010	0.0279	[0.0000; 0.1689]	0.1333
Random effects model	0.0223	[0.0000; 0.1409]	0.1224

Table S11. Leave-one-out meta-analysis of *Ascaris* spp. in captive settings

	proportion	95%-CI	tau^2
Omitting Casim et al., 2015	0.0167	[0.0000; 0.0635]	0.0234
Omitting Dwipayanti et al., 2014	0.0115	[0.0000; 0.0543]	0.0232
Omitting Ferdinandez et al., 2023	0.0162	[0.0000; 0.0624]	0.0236
Omitting Kumalasari et al., 2010	0.0162	[0.0000; 0.0623]	0.0236
Omitting lee et al., 2010	0.0162	[0.0000; 0.0622]	0.0236
Omitting Li et al., 2015	0.0162	[0.0000; 0.0621]	0.0236
Omitting Purnama et al., 2021	0.0161	[0.0000; 0.0620]	0.0236
Omitting Rosyid et al., 2023	0.0083	[0.0000; 0.0420]	0.0172
Omitting Teo et al., 2019	0.0020	[0.0000; 0.0151]	0.0036
Omitting Zanzani et al., 2016	0.0184	[0.0000; 0.0650]	0.0210
Omitting Egorova 2010	0.0161	[0.0000; 0.0643]	0.0241
Random effects model	0.0136	[0.0000; 0.0527]	0.0205
