# Supplementary material for: Prevalence of Soil-Transmitted Helminths in Long-Tailed Macaques (Macaca fascicularis) in Asia: A Systematic Review and Meta-Analysis
Source: Animals (Basel). 2026 Jun 8;16(12):1764. doi: 10.3390/ani16121764 (PMC13295248; doi:10.3390/ani16121764)
Supplement: Supplementary file 1 [file animals-16-01764-s001.zip › Table S1.Search strategies used for each database..pdf]

Table S1. Search strategies used for each database.

General keywords

("*Macaca fascicularis*" OR "long-tailed macaque" OR "cynomolgus macaque" OR macaque*) AND ("soil-transmitted helminth*" OR helminth* OR hookworm* OR Trichuris OR Ascaris OR Strongyloides OR "gastrointestinal parasite*")

PubMed 10 Jan 2026

No.	Key concept	Search terms	Results
1.	Macaca fascicularis	(“Macaca fascicularis"[MeSH Terms] OR "Macaca fascicularis"[Title/Abstract] OR "long-tailed macaque"[Title/Abstract] OR "long tailed macaque"[Title/Abstract] OR "crab-eating macaque"[Title/Abstract] OR "cynomolgus macaque"[Title/Abstract] OR macaque*[Title/Abstract] OR monkey*[Title/Abstract])	128,312
2.	Soil-transmitted helminth OR Gastrointestinal parasite	("gastrointestinal parasite*"[Title/Abstract] OR "GI parasite*"[Title/Abstract] OR endoparasite*[Title/Abstract] OR "intestinal parasite*"[Title/Abstract] OR "soil-transmitted helminth*"[Title/Abstract] OR "soil transmitted helminth*"[Title/Abstract] OR STH[Title/Abstract] OR STHs [Title/Abstract] OR helminth*[Title/Abstract] OR hookworm*[Title/Abstract] OR Trichuris [Title/Abstract] OR Ascaris [Title/Abstract] OR Strongyloides[Title/Abstract])	46,414
3.	1 AND 2	#1 AND #2	387

Embase 10 Jan 2026

No.	Key concept	Search terms	Results
1.	Macaca fascicularis	('macaca fascicularis'/exp OR 'macaca fascicularis':ti,ab OR 'long-tailed macaque':ti,ab OR 'long tailed macaque':ti,ab OR 'crab-eating macaque':ti,ab OR 'cynomolgus macaque':ti,ab OR macaque*: ti,ab OR monkey*:ti,ab)	150,447
2.	Soil-transmitted helminth OR Gastrointestinal parasite	('gastrointestinal parasite*':ti,ab OR 'gi parasite*':ti,ab OR endoparasite*:ti,ab OR 'intestinal parasite*':ti,ab	52,613

		OR 'soil-transmitted helminth*':ti,ab<br>OR 'soil transmitted helminth*':ti,ab<br>OR sth:ti,ab<br>OR sths:ti,ab<br>OR helminth*:ti,ab<br>OR hookworm*:ti,ab<br>OR trichuris:ti,ab<br>OR ascaris:ti,ab<br>OR strongyloides:ti,ab)	
3.	1 AND 2	#1 AND #2	417

Scopus 10 Jan 2026

No.	Key concept	Search terms	Results
1.	Macaca fascicularis	(TITLE-ABS-KEY("Macaca fascicularis")<br>OR TITLE-ABS-KEY("long-tailed macaque")<br>OR TITLE-ABS-KEY("long tailed macaque")<br>OR TITLE-ABS-KEY("crab-eating macaque")<br>OR TITLE-ABS-KEY("cynomolgus macaque")<br>OR TITLE-ABS-KEY(macaque*)<br>OR TITLE-ABS-KEY(monkey*))	181,442
2.	Soil-transmitted helminth OR Gastrointestinal parasite	(TITLE-ABS-KEY("gastrointestinal parasite*")<br>OR TITLE-ABS-KEY("GI parasite*")<br>OR TITLE-ABS-KEY(endoparasite*)<br>OR TITLE-ABS-KEY("intestinal parasite*")<br>OR TITLE-ABS-KEY("soil-transmitted helminth*")<br>OR TITLE-ABS-KEY("soil transmitted helminth*")<br>OR TITLE-ABS-KEY(STH)<br>OR TITLE-ABS-KEY(STHs)<br>OR TITLE-ABS-KEY(helminth*)<br>OR TITLE-ABS-KEY(hookworm*)<br>OR TITLE-ABS-KEY(Trichuris)<br>OR TITLE-ABS-KEY(Ascaris)<br>OR TITLE-ABS-KEY(Strongyloides))	97,775
3.	1 AND 2	#1 AND #2	785

Google Scholar 10 Jan 2026

No.	Key concept	Search terms	Results
1.	Macaca fascicularis AND (Soil-transmitted helminth OR Gastrointestinal parasite)	Macaca fascicularis AND ("soil-transmitted helminths" OR "gastrointestinal parasites")	570
