# Supplementary material for: Prevalence of Soil-Transmitted Helminths in Long-Tailed Macaques (Macaca fascicularis) in Asia: A Systematic Review and Meta-Analysis
Source: Animals (Basel). 2026 Jun 8;16(12):1764. doi: 10.3390/ani16121764 (PMC13295248; doi:10.3390/ani16121764)

1. Pooled prevalence of *Strongyloides* spp.in captive long-tailed macaque.

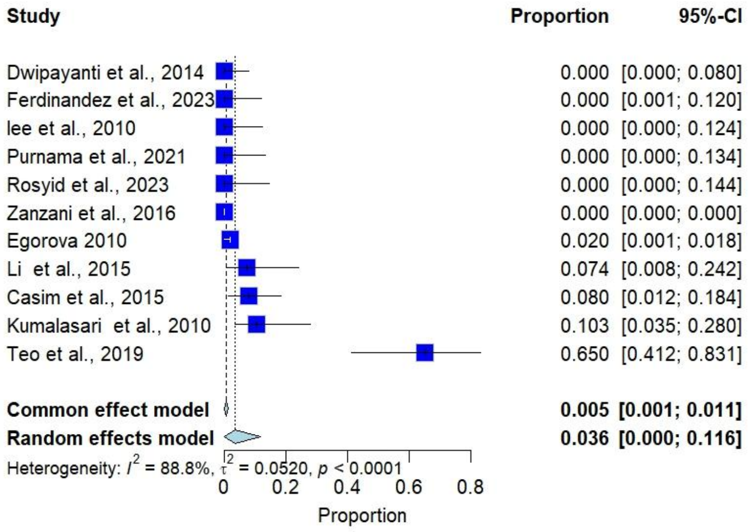

2. Pooled prevalence of *Trichuris* spp.in captive long-tailed macaque.

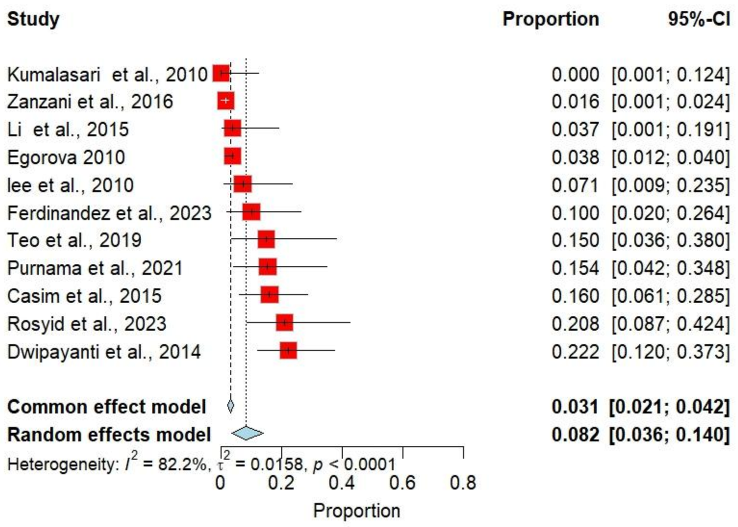

3. Pooled prevalence of hookworm in captive long-tailed macaque.

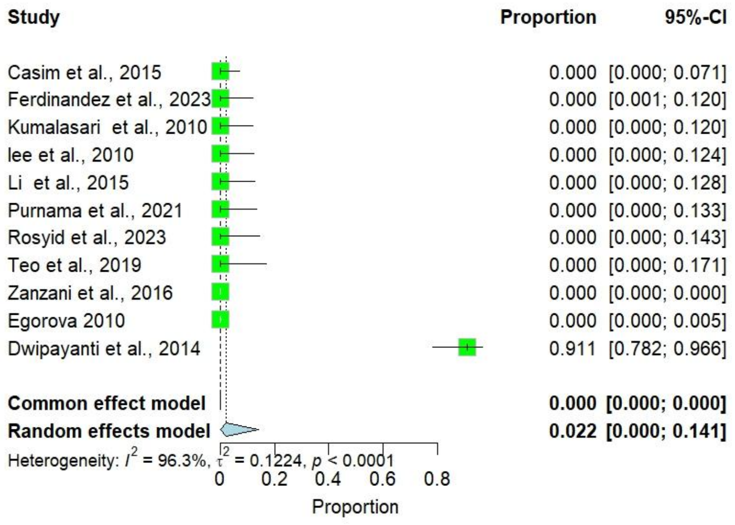

4. Pooled prevalence of *Ascaris* spp.in captive long-tailed macaque.

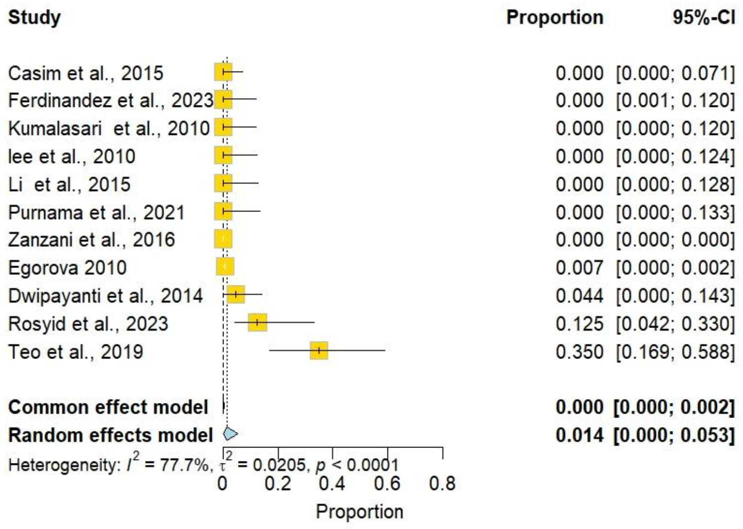

Supplement: Supplementary file 1 [file animals-16-01764-s001.zip › Supplementary file S1. Pooled prevalence of STHs in captive long-tailed macaque.pdf]
