# Supplementary material for: Prevalence of Soil-Transmitted Helminths in Long-Tailed Macaques (Macaca fascicularis) in Asia: A Systematic Review and Meta-Analysis
Source: Animals (Basel). 2026 Jun 8;16(12):1764. doi: 10.3390/ani16121764 (PMC13295248; doi:10.3390/ani16121764)

1. Subgroup Analysis of *Strongyloides* spp. Infections in Free-Ranging Long-Tailed Macaques by Country

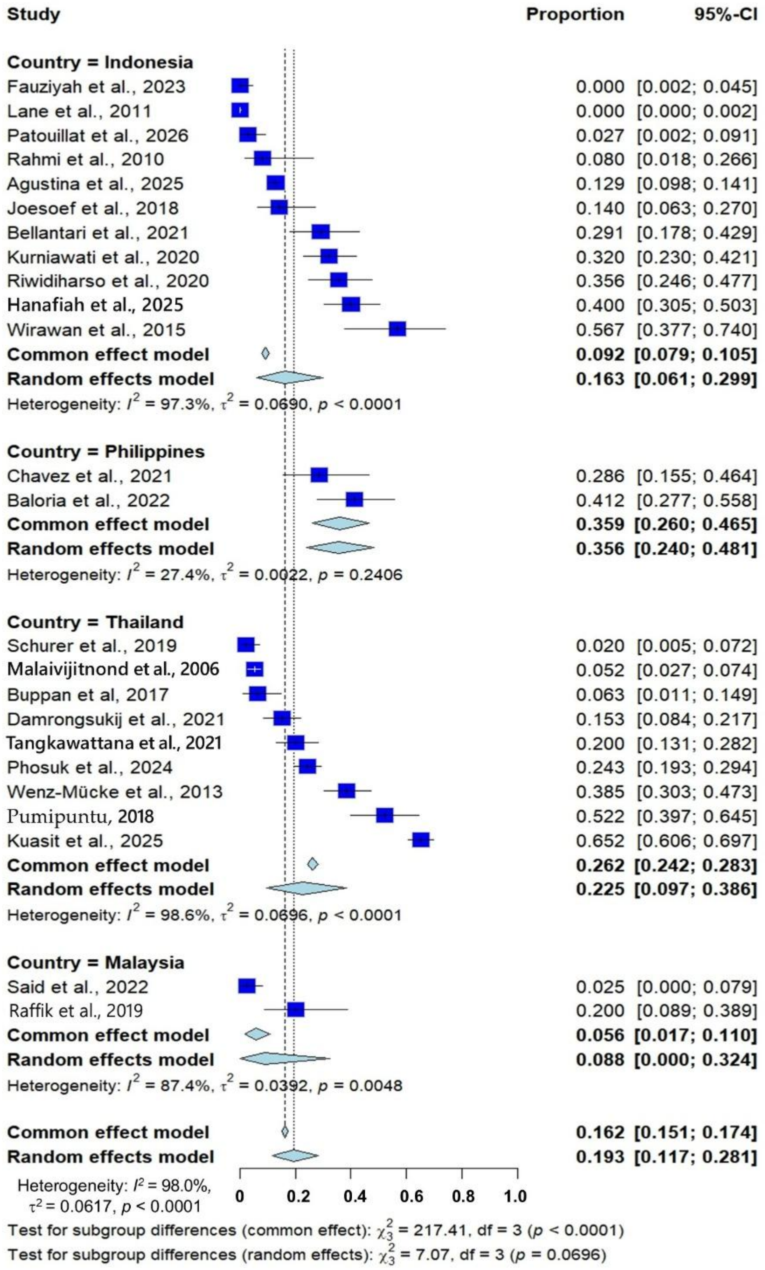

2. Subgroup Analysis of *Trichuris* spp. Infections in Free-Ranging Long-Tailed Macaques by Country

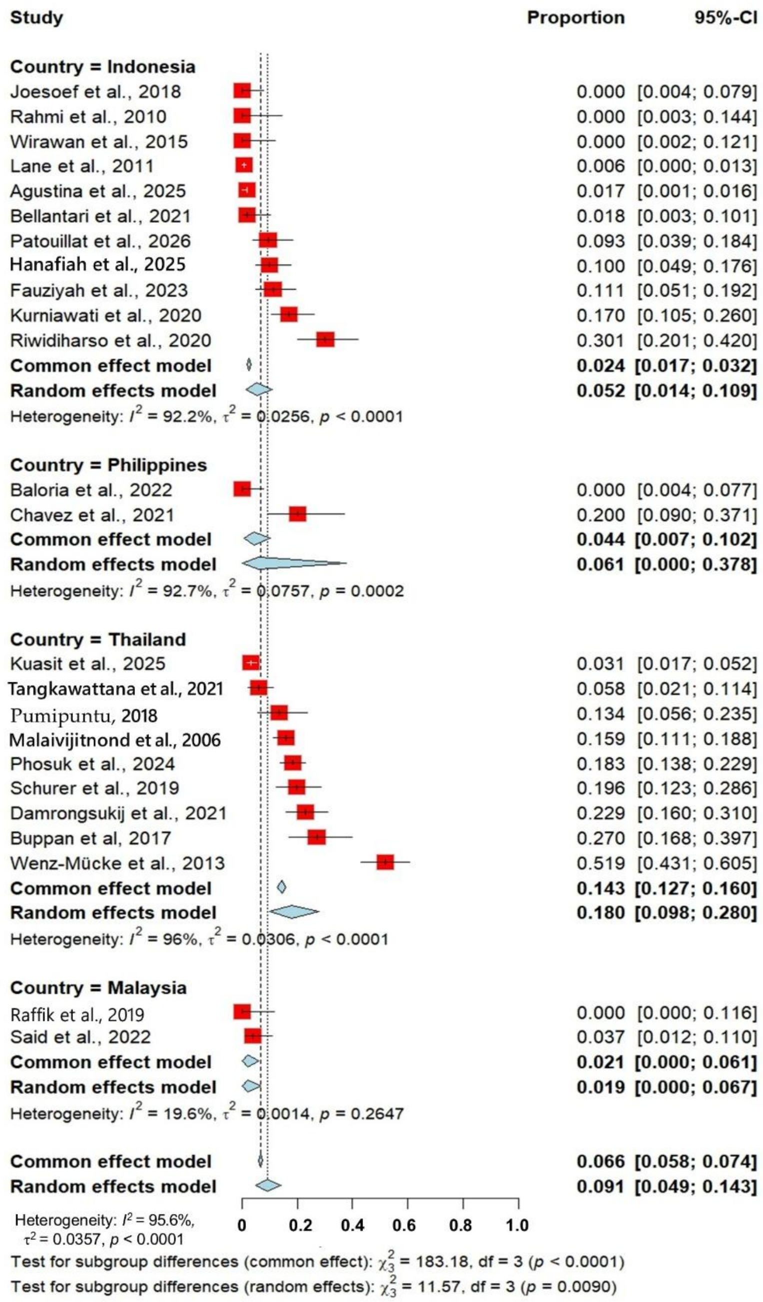

3. Subgroup Analysis of Hookworm Infections in Free-Ranging Long-Tailed Macaques by Country

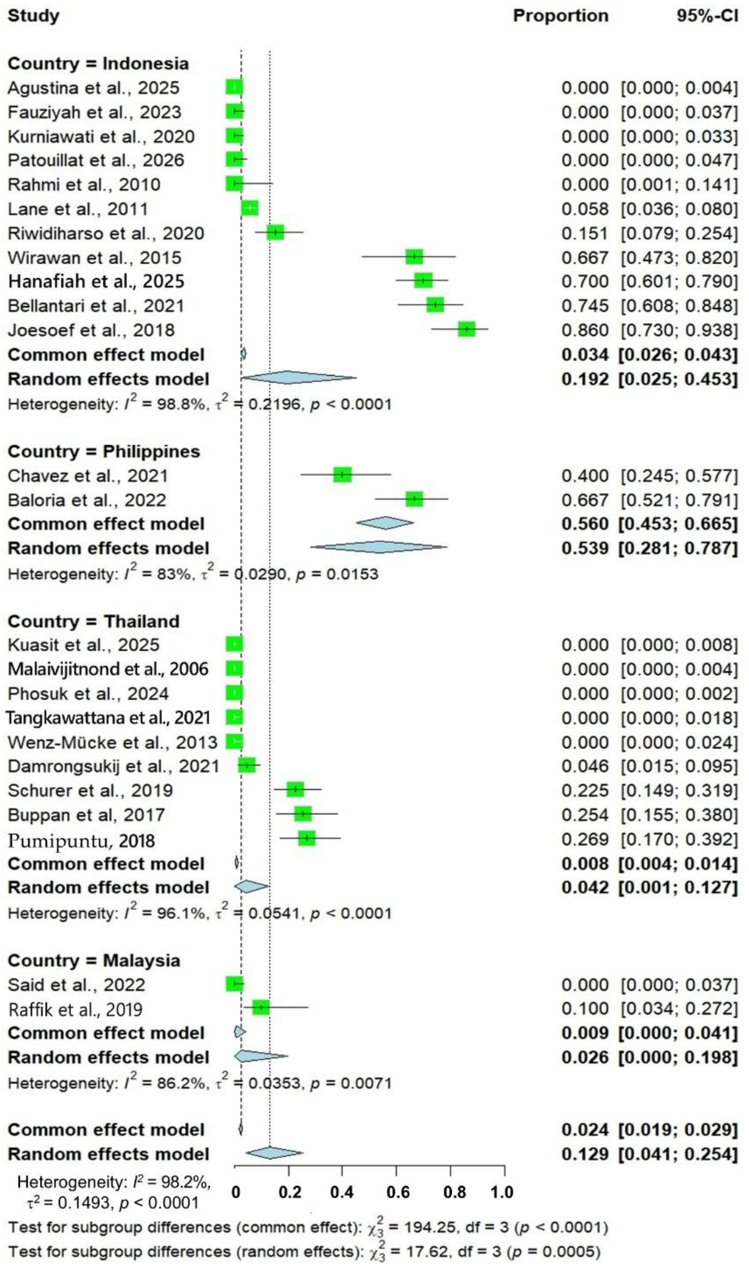

4. Subgroup Analysis of *Ascaris* spp. Infections in Free-Ranging Long-Tailed Macaques by Country

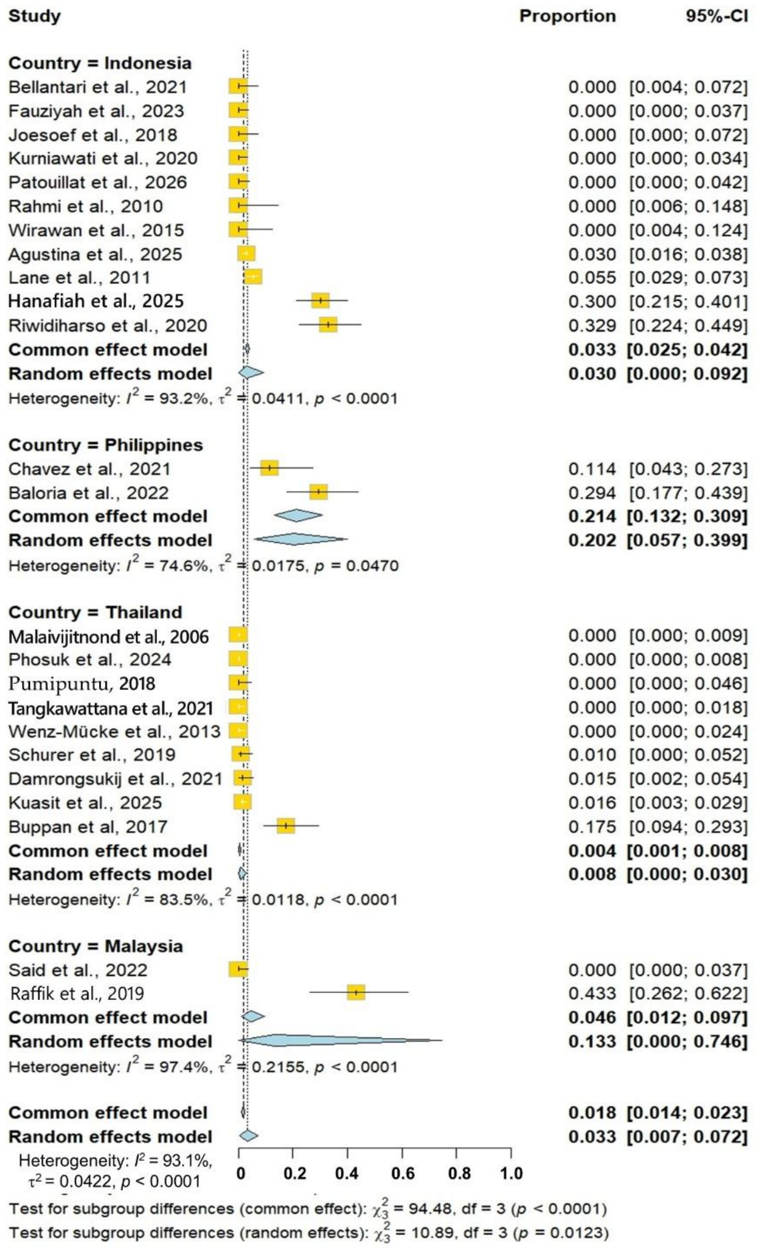

5. Subgroup Analysis of *Strongyloides* spp. Infections in Free-Ranging Long-Tailed Macaques by Habitat

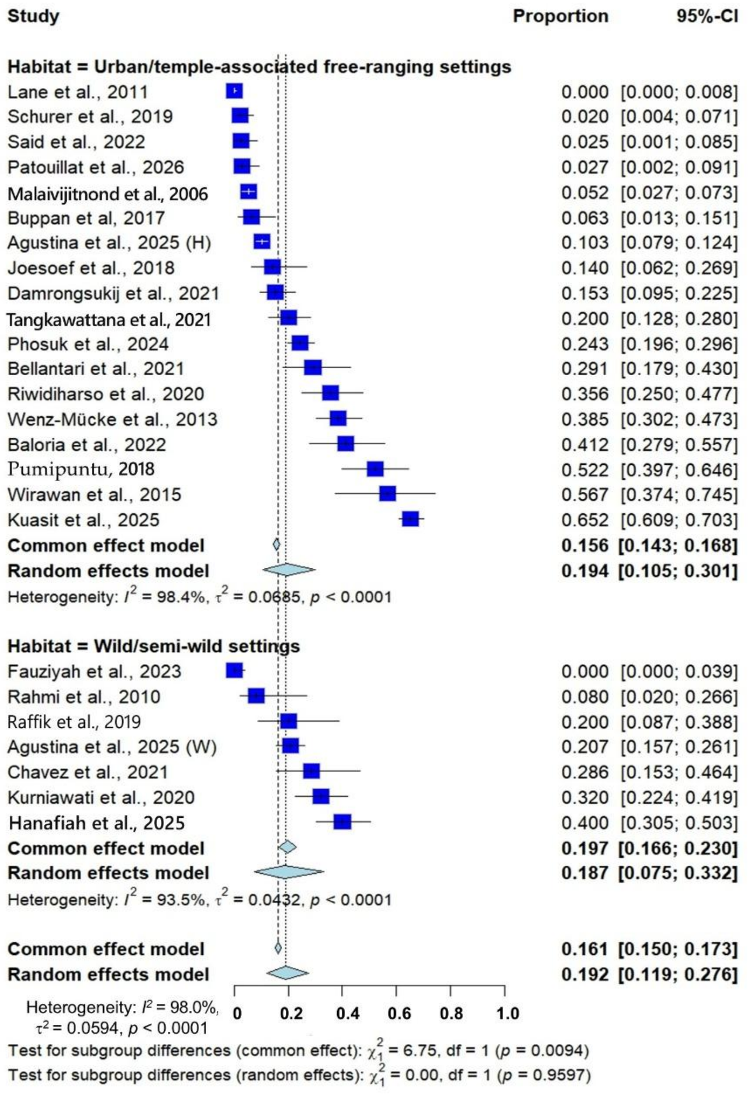

6. Subgroup Analysis of *Trichuris* spp. Infections in Free-Ranging Long-Tailed Macaques by Habitat

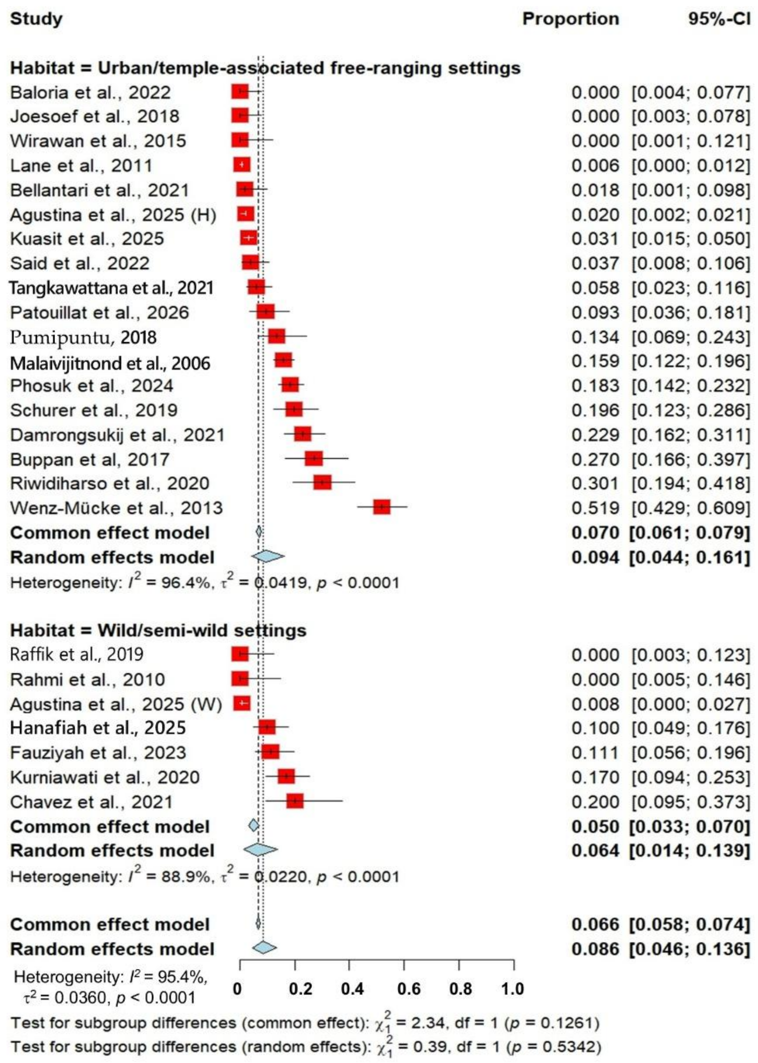

7. Subgroup Analysis of Hookworm Infections in Free-Ranging Long-Tailed Macaques by Habitat

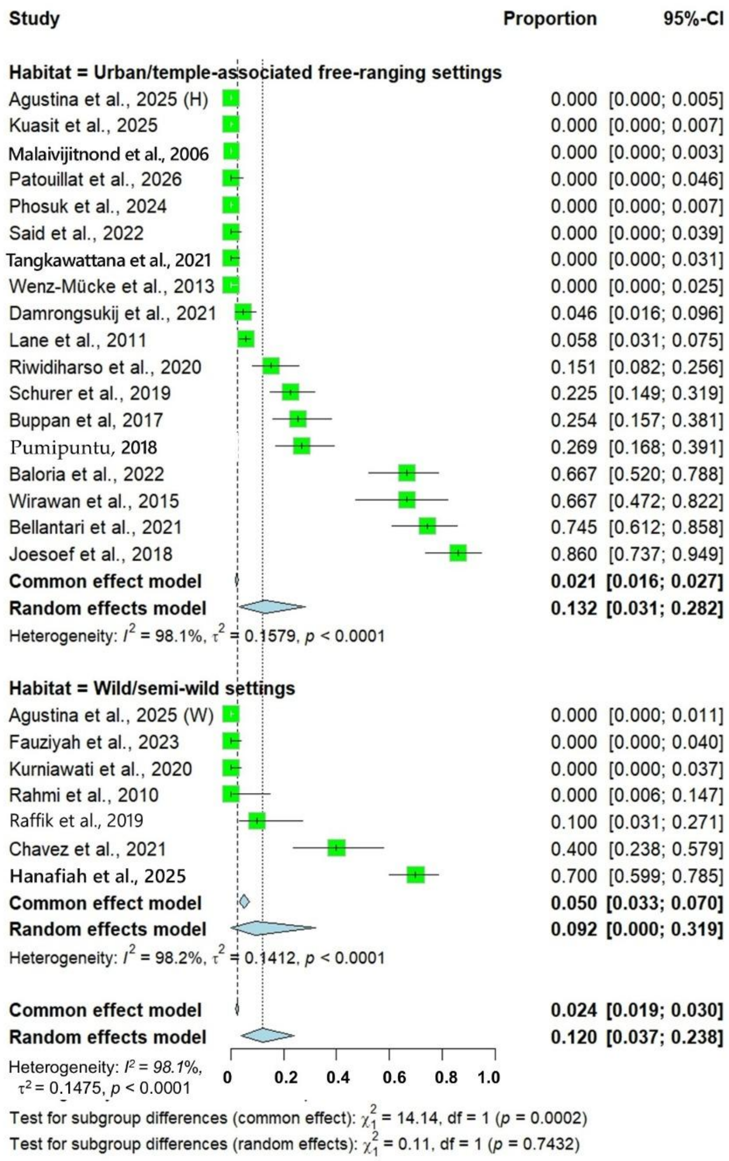

8. Subgroup Analysis of *Ascaris spp.* Infections in Free-Ranging Long-Tailed Macaques by Habitat

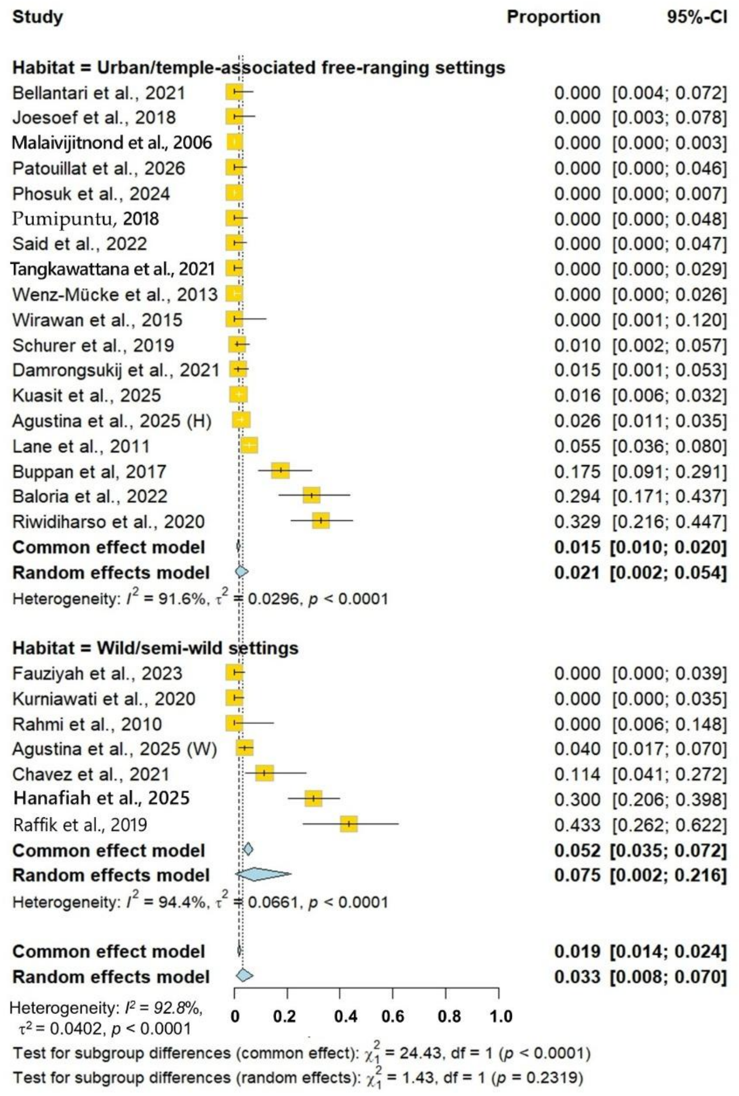

9. Subgroup Analysis of *Strongyloides* spp. Infections in Free-Ranging Long-Tailed Macaques by Diagnostic Method

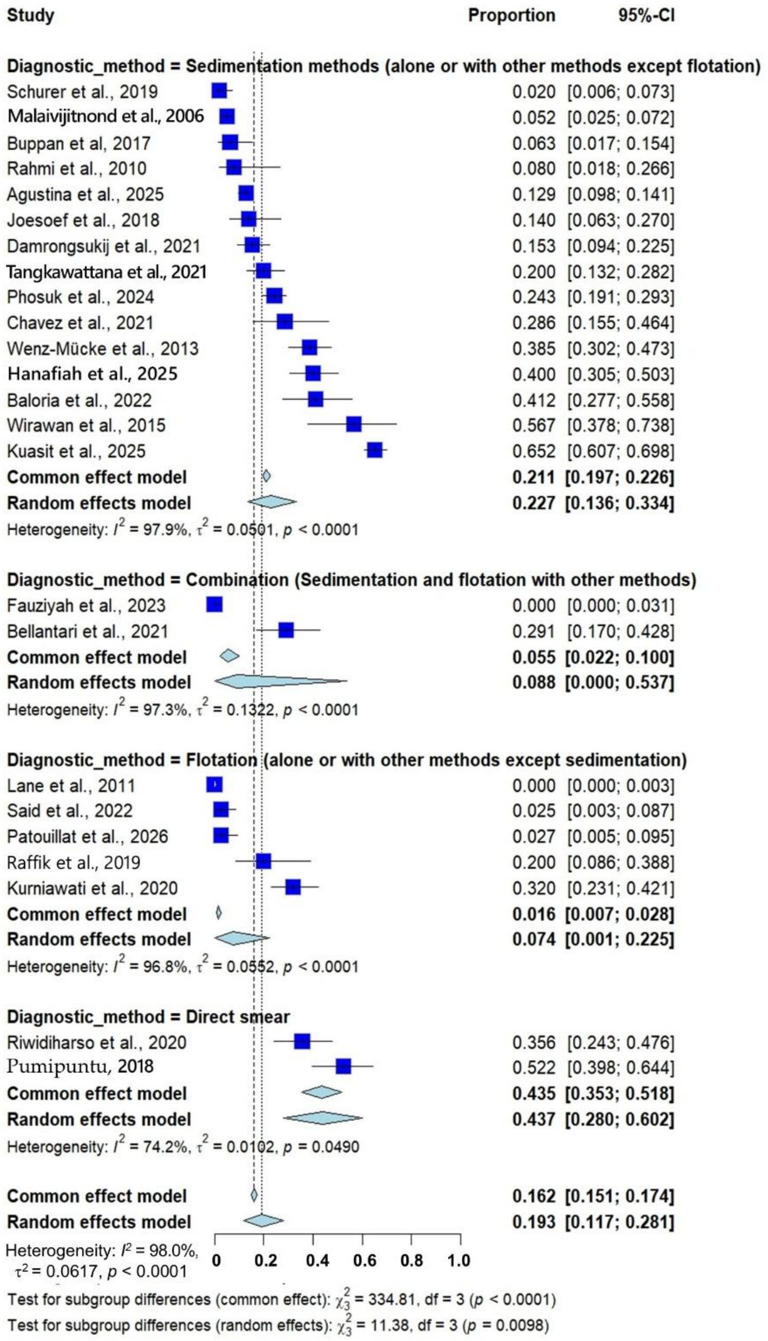

10. Subgroup Analysis of *Trichuris* spp. Infections in Free-Ranging Long-Tailed Macaques by Diagnostic Method

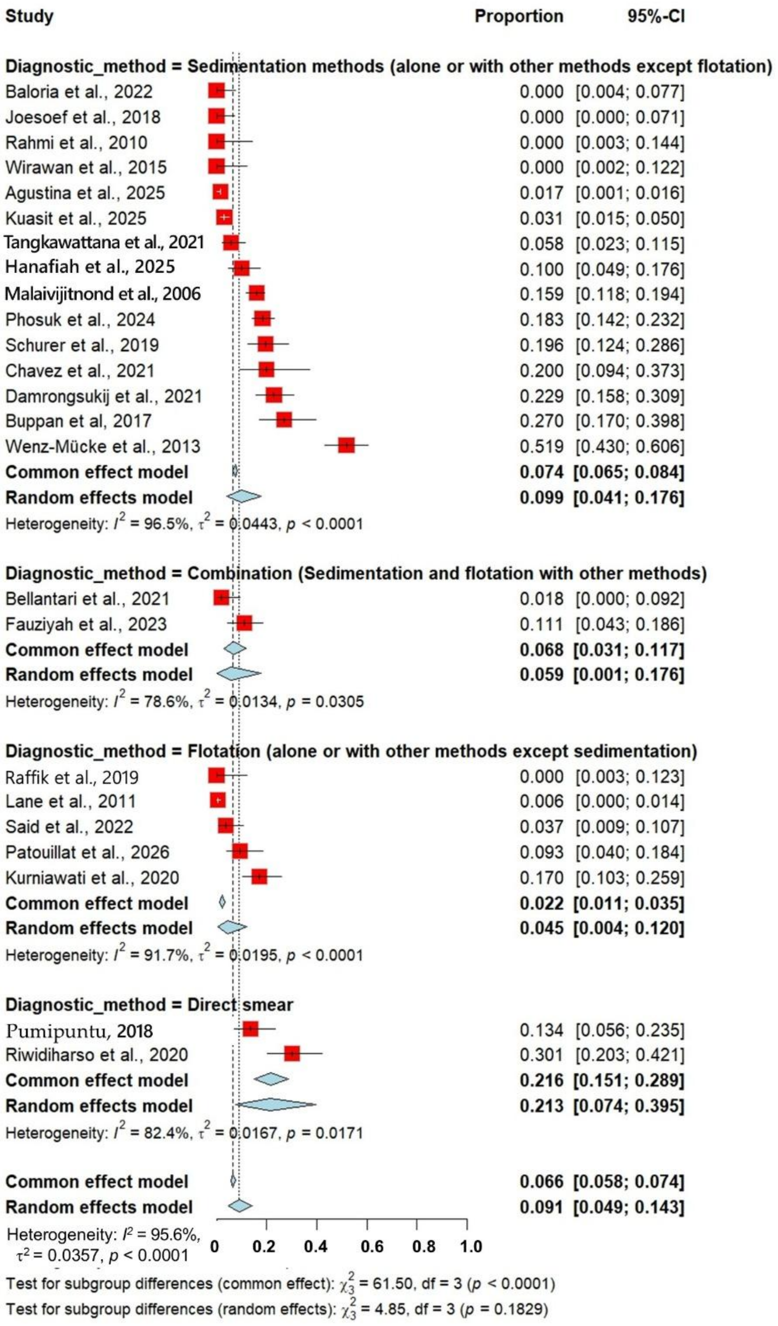

11. Subgroup Analysis of Hookworm Infections in Free-Ranging Long-Tailed Macaques by Diagnostic Method

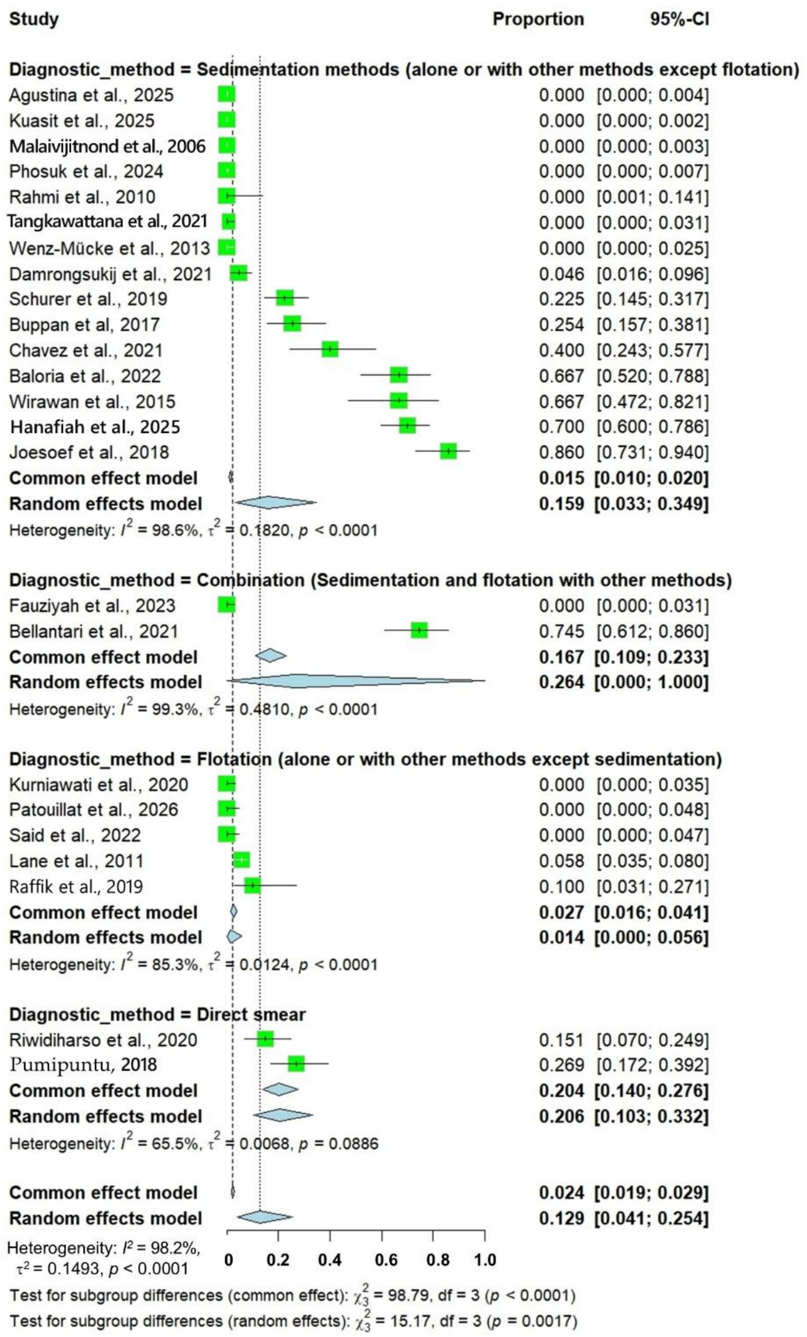

12. Subgroup Analysis of *Ascaris* spp. Infections in Free-Ranging Long-Tailed Macaques by Diagnostic Method

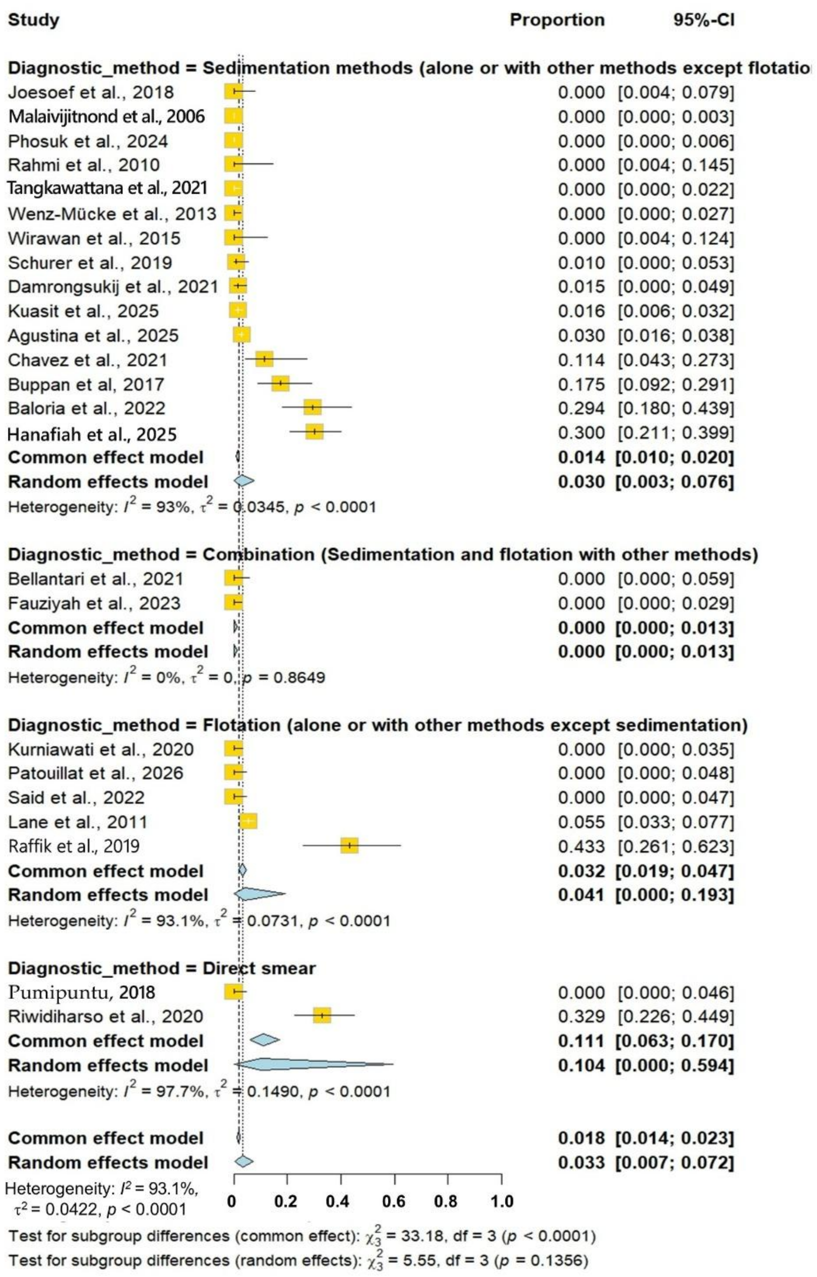

Supplement: Supplementary file 1 [file animals-16-01764-s001.zip › Supplementary file S2. Subgroup analysis of STHs_edited.pdf]
