# Supplementary material for: Prevalence of Soil-Transmitted Helminths in Long-Tailed Macaques (Macaca fascicularis) in Asia: A Systematic Review and Meta-Analysis
Source: Animals (Basel). 2026 Jun 8;16(12):1764. doi: 10.3390/ani16121764 (PMC13295248; doi:10.3390/ani16121764)
Supplement: Supplementary file 1 [file animals-16-01764-s001.zip › Supplementary file S3. meta-regression.pdf]

1. *Strongyloides* spp.

1.1. Meta-regression country group (Thailand, Indonesia and other countries)

```
Mixed-Effects Model (k = 24; tau^2 estimator: REML)

tau^2 (estimated amount of residual heterogeneity):     2.1526 (SE = 0.7329)
tau (square root of estimated tau^2 value):             1.4672
I^2 (residual heterogeneity / unaccounted variability): 97.37%
H^2 (unaccounted variability / sampling variability):   37.98
R^2 (amount of heterogeneity accounted for):            0.00%

Test for Residual Heterogeneity:
QE(df = 21) = 502.2709, p-val < .0001

Test of Moderators (coefficients 2:3):
F(df1 = 2, df2 = 21) = 0.1355, p-val = 0.8740

Model Results:

                      estimate      se     tval  df    pval    ci.lb
intrcpt                -1.4335  0.5413  -2.6483  21  0.0150  -2.5591
Country_NewIndonesia   -0.3745  0.7484  -0.5004  21  0.6220  -1.9310
Country_NewOthers      -0.0648  0.9931  -0.0652  21  0.9486  -2.1301
                        ci.ub
intrcpt               -0.3078  *
Country_NewIndonesia   1.1819
Country_NewOthers      2.0005

---
Signif. codes:  0 ‘***’ 0.001 ‘**’ 0.01 ‘*’ 0.05 ‘.’ 0.1 ‘ ’ 1
```

1.2. Meta-regression diagnostic method (Sedimentation, Flotation and other)

```
Mixed-Effects Model (k = 24; tau^2 estimator: REML)

tau^2 (estimated amount of residual heterogeneity):     1.7808 (SE = 0.6135)
tau (square root of estimated tau^2 value):             1.3345
I^2 (residual heterogeneity / unaccounted variability): 97.12%
H^2 (unaccounted variability / sampling variability):   34.74
R^2 (amount of heterogeneity accounted for):            4.92%

Test for Residual Heterogeneity:
QE(df = 21) = 575.7958, p-val < .0001

Test of Moderators (coefficients 2:3):
F(df1 = 2, df2 = 21) = 1.6865, p-val = 0.2093

Model Results:

                     estimate      se     tval  df    pval    ci.lb
intrcpt               -1.3535  0.3864  -3.5032  21  0.0021  -2.1571
Method_NewFlotation   -1.4292  0.8281  -1.7259  21  0.0991  -3.1514
Method_NewOthers       0.2288  0.8783   0.2604  21  0.7971  -1.5978
                       ci.ub
intrcpt              -0.5500  **
Method_NewFlotation   0.2930   .
Method_NewOthers      2.0553

---
Signif. codes:  0 ‘***’ 0.001 ‘**’ 0.01 ‘*’ 0.05 ‘.’ 0.1 ‘ ’ 1
```

1.3. Meta-regression habitat (urban/temple and wild/semi-wild)

```
Mixed-Effects Model (k = 25; tau^2 estimator: REML)

  logLik  deviance       AIC       BIC      AICc
-43.6717   87.3434   93.3434   96.7499   94.6066

tau^2 (estimated amount of residual heterogeneity):     1.8747 (SE = 0.6128)
tau (square root of estimated tau^2 value):             1.3692
I^2 (residual heterogeneity / unaccounted variability): 97.13%
H^2 (unaccounted variability / sampling variability):   34.79
R^2 (amount of heterogeneity accounted for):            0.00%

Test for Residual Heterogeneity:
QE(df = 23) = 593.2203, p-val < .0001

Test of Moderators (coefficient 2):
F(df1 = 1, df2 = 23) = 0.0380, p-val = 0.8472

Model Results:

                       estimate      se     tval  df    pval    ci.lb
intrcpt                 -1.6252  0.3718  -4.3715  23  0.0002  -2.3943
HabitatWild/semi-wild    0.1394  0.7154   0.1949  23  0.8472  -1.3404
                         ci.ub
intrcpt                -0.8561  ***
HabitatWild/semi-wild   1.6192

---
Signif. codes:  0 ‘***’ 0.001 ‘**’ 0.01 ‘*’ 0.05 ‘.’ 0.1 ‘ ’ 1
```

2. *Trichuris* spp.

2.1. Meta-regression country group (Thailand, Indonesia and other countries)

```
Mixed-Effects Model (k = 24; tau^2 estimator: REML)

tau^2 (estimated amount of residual heterogeneity):     1.3181 (SE = 0.4931)
tau (square root of estimated tau^2 value):             1.1481
I^2 (residual heterogeneity / unaccounted variability): 93.93%
H^2 (unaccounted variability / sampling variability):   16.47
R^2 (amount of heterogeneity accounted for):            11.80%

Test for Residual Heterogeneity:
QE(df = 21) = 284.7376, p-val < .0001

Test of Moderators (coefficients 2:3):
F(df1 = 2, df2 = 21) = 3.0125, p-val = 0.0708

Model Results:

                       estimate      se     tval  df    pval    ci.lb
intrcpt                 -2.8788  0.3940  -7.3068  21  <.0001  -3.6982
Country_groupOther      -0.0625  0.8176  -0.0764  21  0.9398  -1.7628
Country_groupThailand    1.2523  0.5501   2.2763  21  0.0334   0.1082
                         ci.ub
intrcpt                -2.0595  ***
Country_groupOther      1.6379
Country_groupThailand   2.3964    *

---
Signif. codes:  0 ‘***’ 0.001 ‘**’ 0.01 ‘*’ 0.05 ‘.’ 0.1 ‘ ’ 1
```

2.2. <u>Meta-regression diagnostic method (Sedimentation, Flotation and other)</u>

```
Mixed-Effects Model (k = 24; tau^2 estimator: REML)

tau^2 (estimated amount of residual heterogeneity):     1.4959 (SE = 0.5544)
tau (square root of estimated tau^2 value):             1.2231
I^2 (residual heterogeneity / unaccounted variability): 94.57%
H^2 (unaccounted variability / sampling variability):   18.40
R^2 (amount of heterogeneity accounted for):            0.00%

Test for Residual Heterogeneity:
QE(df = 21) = 328.7431, p-val < .0001

Test of Moderators (coefficients 2:3):
F(df1 = 2, df2 = 21) = 0.9022, p-val = 0.4208

Model Results:

                     estimate      se     tval  df    pval    ci.lb
intrcpt               -2.2047  0.3553  -6.2049  21  <.0001  -2.9436
MethodFlotation       -0.8805  0.7206  -1.2219  21  0.2353  -2.3791
MethodOther methods    0.1987  0.7602   0.2614  21  0.7963  -1.3822
                       ci.ub
intrcpt              -1.4658  ***
MethodFlotation       0.6181
MethodOther methods   1.7797

---
Signif. codes:  0 ‘***’ 0.001 ‘**’ 0.01 ‘*’ 0.05 ‘.’ 0.1 ‘ ’ 1
```

2.3. <u>Meta-regression habitat (urban/temple and wild/semi-wild)</u>

```
Mixed-Effects Model (k = 25; tau^2 estimator: REML)

  logLik  deviance       AIC       BIC      AICc
-41.6240   83.2481   89.2481   92.6545   90.5112

tau^2 (estimated amount of residual heterogeneity):     1.7170 (SE = 0.5999)
tau (square root of estimated tau^2 value):             1.3103
I^2 (residual heterogeneity / unaccounted variability): 95.05%
H^2 (unaccounted variability / sampling variability):   20.20
R^2 (amount of heterogeneity accounted for):            0.00%

Test for Residual Heterogeneity:
QE(df = 23) = 334.2567, p-val < .0001

Test of Moderators (coefficient 2):
F(df1 = 1, df2 = 23) = 0.1130, p-val = 0.7398

Model Results:

                       estimate      se     tval  df    pval    ci.lb
intrcpt                 -2.3934  0.3378  -7.0843  23  <.0001  -3.0923
HabitatWild/semi-wild   -0.2213  0.6581  -0.3362  23  0.7398  -1.5826
                         ci.ub
intrcpt                -1.6945  ***
HabitatWild/semi-wild   1.1401

---
Signif. codes:  0 ‘***’ 0.001 ‘**’ 0.01 ‘*’ 0.05 ‘.’ 0.1 ‘ ’ 1
```

3. Hookworm

3.1. Meta-regression country group (Thailand, Indonesia and other countries)

```
Mixed-Effects Model (k = 24; tau^2 estimator: REML)

tau^2 (estimated amount of residual heterogeneity):     7.1924 (SE = 2.4838)
tau (square root of estimated tau^2 value):             2.6819
I^2 (residual heterogeneity / unaccounted variability): 97.56%
H^2 (unaccounted variability / sampling variability):   40.94
R^2 (amount of heterogeneity accounted for):            2.40%

Test for Residual Heterogeneity:
QE(df = 21) = 422.1952, p-val < .0001

Test of Moderators (coefficients 2:3):
F(df1 = 2, df2 = 21) = 1.2753, p-val = 0.3001

Model Results:

                       estimate      se     tval  df    pval    ci.lb
intrcpt                 -2.1658  0.8597  -2.5194  21  0.0199  -3.9536
Country_groupOther       0.6069  1.6428   0.3694  21  0.7155  -2.8095
Country_groupThailand   -1.7116  1.2896  -1.3273  21  0.1987  -4.3934
                         ci.ub
intrcpt                -0.3781  *
Country_groupOther      4.0234
Country_groupThailand   0.9702

---
Signif. codes:  0 ‘***’ 0.001 ‘**’ 0.01 ‘*’ 0.05 ‘.’ 0.1 ‘ ’ 1
```

3.2. Meta-regression diagnostic method (Sedimentation, Flotation and other)

```
Mixed-Effects Model (k = 24; tau^2 estimator: REML)

tau^2 (estimated amount of residual heterogeneity):     7.4933 (SE = 2.5770)
tau (square root of estimated tau^2 value):             2.7374
I^2 (residual heterogeneity / unaccounted variability): 97.50%
H^2 (unaccounted variability / sampling variability):   39.94
R^2 (amount of heterogeneity accounted for):            0.00%

Test for Residual Heterogeneity:
QE(df = 21) = 320.7994, p-val < .0001

Test of Moderators (coefficients 2:3):
F(df1 = 2, df2 = 21) = 0.7825, p-val = 0.4701

Model Results:

                     estimate      se     tval  df    pval    ci.lb
intrcpt               -2.6266  0.7536  -3.4851  21  0.0022  -4.1939
MethodFlotation       -1.3191  1.5275  -0.8636  21  0.3976  -4.4958
MethodOther methods    1.0982  1.6116   0.6814  21  0.5030  -2.2533
                       ci.ub
intrcpt              -1.0593  **
MethodFlotation       1.8575
MethodOther methods   4.4497

---
Signif. codes:  0 ‘***’ 0.001 ‘**’ 0.01 ‘*’ 0.05 ‘.’ 0.1 ‘ ’ 1
```

3.3. Meta-regression habitat (urban/temple and wild/semi-wild)

```
Mixed-Effects Model (k = 25; tau^2 estimator: REML)

tau^2 (estimated amount of residual heterogeneity):     7.7899 (SE = 2.5581)
tau (square root of estimated tau^2 value):             2.7910
I^2 (residual heterogeneity / unaccounted variability): 97.72%
H^2 (unaccounted variability / sampling variability):   43.87
R^2 (amount of heterogeneity accounted for):            0.00%

Test for Residual Heterogeneity:
QE(df = 23) = 440.6846, p-val < .0001

Test of Moderators (coefficient 2):
F(df1 = 1, df2 = 23) = 0.0189, p-val = 0.8919

Model Results:

                       estimate      se     tval  df    pval    ci.lb
intrcpt                 -2.7739  0.6939  -3.9973  23  0.0006  -4.2094
HabitatWild/semi-wild   -0.1824  1.3268  -0.1375  23  0.8919  -2.9271
                          ci.ub
intrcpt                 -1.3384  ***
HabitatWild/semi-wild    2.5624

---
Signif. codes:  0 ‘***’ 0.001 ‘**’ 0.01 ‘*’ 0.05 ‘.’ 0.1 ‘ ’ 1
```

4. *Ascaris* spp.

4.1. Meta-regression country group (Thailand, Indonesia and other countries)

```
Mixed-Effects Model (k = 24; tau^2 estimator: REML)

tau^2 (estimated amount of residual heterogeneity):     2.2958 (SE = 0.9779)
tau (square root of estimated tau^2 value):             1.5152
I^2 (residual heterogeneity / unaccounted variability): 91.64%
H^2 (unaccounted variability / sampling variability):   11.97
R^2 (amount of heterogeneity accounted for):            25.24%

Test for Residual Heterogeneity:
QE(df = 21) = 222.4574, p-val < .0001

Test of Moderators (coefficients 2:3):
F(df1 = 2, df2 = 21) = 4.1929, p-val = 0.0294

Model Results:

                       estimate      se     tval  df    pval    ci.lb
intrcpt                 -3.3022  0.5227  -6.3177  21  <.0001  -4.3892
Country_groupOther       1.6119  0.9491   1.6984  21  0.1042  -0.3618
Country_groupThailand   -1.2252  0.7855  -1.5598  21  0.1337  -2.8586
                          ci.ub
intrcpt                 -2.2152  ***
Country_groupOther       3.5856
Country_groupThailand    0.4083

---
Signif. codes:  0 ‘***’ 0.001 ‘**’ 0.01 ‘*’ 0.05 ‘.’ 0.1 ‘ ’ 1
```

4.2. Meta-regression diagnostic method (Sedimentation, Flotation and other)

```
Mixed-Effects Model (k = 24; tau^2 estimator: REML)

tau^2 (estimated amount of residual heterogeneity):     3.3863 (SE = 1.3342)
tau (square root of estimated tau^2 value):             1.8402
I^2 (residual heterogeneity / unaccounted variability): 93.22%
H^2 (unaccounted variability / sampling variability):   14.75
R^2 (amount of heterogeneity accounted for):            0.00%

Test for Residual Heterogeneity:
QE(df = 21) = 254.0558, p-val < .0001

Test of Moderators (coefficients 2:3):
F(df1 = 2, df2 = 21) = 0.0418, p-val = 0.9592

Model Results:

                     estimate      se     tval  df    pval    ci.lb
intrcpt               -3.6169  0.5150  -7.0230  21  <.0001  -4.6880
MethodFlotation        0.2967  1.0411   0.2850  21  0.7785  -1.8685
MethodOther methods    0.1279  1.1666   0.1097  21  0.9137  -2.2981
                       ci.ub
intrcpt              -2.5459  ***
MethodFlotation       2.4618
MethodOther methods   2.5540

---
Signif. codes:  0 ‘***’ 0.001 ‘**’ 0.01 ‘*’ 0.05 ‘.’ 0.1 ‘ ’ 1

> predict(reg_ascaris, transf = transf.ilogit, digits = 4)
```

4.3. Meta-regression habitat (urban/temple and wild/semi-wild)

```
Mixed-Effects Model (k = 25; tau^2 estimator: REML)

tau^2 (estimated amount of residual heterogeneity):     2.7015 (SE = 1.0460)
tau (square root of estimated tau^2 value):             1.6436
I^2 (residual heterogeneity / unaccounted variability): 92.69%
H^2 (unaccounted variability / sampling variability):   13.67
R^2 (amount of heterogeneity accounted for):            6.41%

Test for Residual Heterogeneity:
QE(df = 23) = 248.3661, p-val < .0001

Test of Moderators (coefficient 2):
F(df1 = 1, df2 = 23) = 2.4307, p-val = 0.1326

Model Results:

                       estimate      se     tval  df    pval    ci.lb
intrcpt                 -3.8355  0.4365  -8.7866  23  <.0001  -4.7385
HabitatWild/semi-wild    1.2506  0.8022   1.5591  23  0.1326  -0.4088
                         ci.ub
intrcpt                -2.9325  ***
HabitatWild/semi-wild   2.9100

---
Signif. codes:  0 ‘***’ 0.001 ‘**’ 0.01 ‘*’ 0.05 ‘.’ 0.1 ‘ ’ 1
```
