# Supplementary material for: Prevalence of Soil-Transmitted Helminths in Long-Tailed Macaques (Macaca fascicularis) in Asia: A Systematic Review and Meta-Analysis
Source: Animals (Basel). 2026 Jun 8;16(12):1764. doi: 10.3390/ani16121764 (PMC13295248; doi:10.3390/ani16121764)

1. Sensitivity analysis including the excluded small-sample study for *Strongyloides* spp. prevalence.

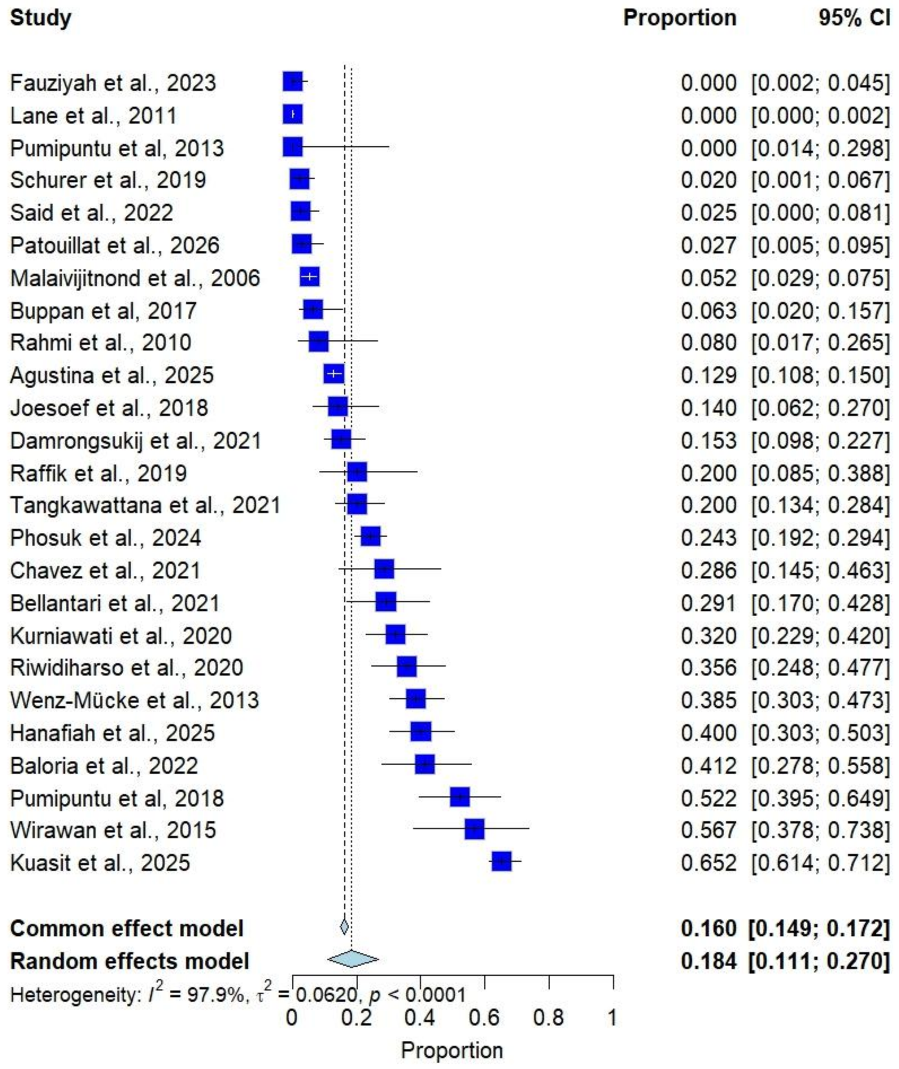

2. Sensitivity analysis including the excluded small-sample study for *Trichuris* spp. prevalence.

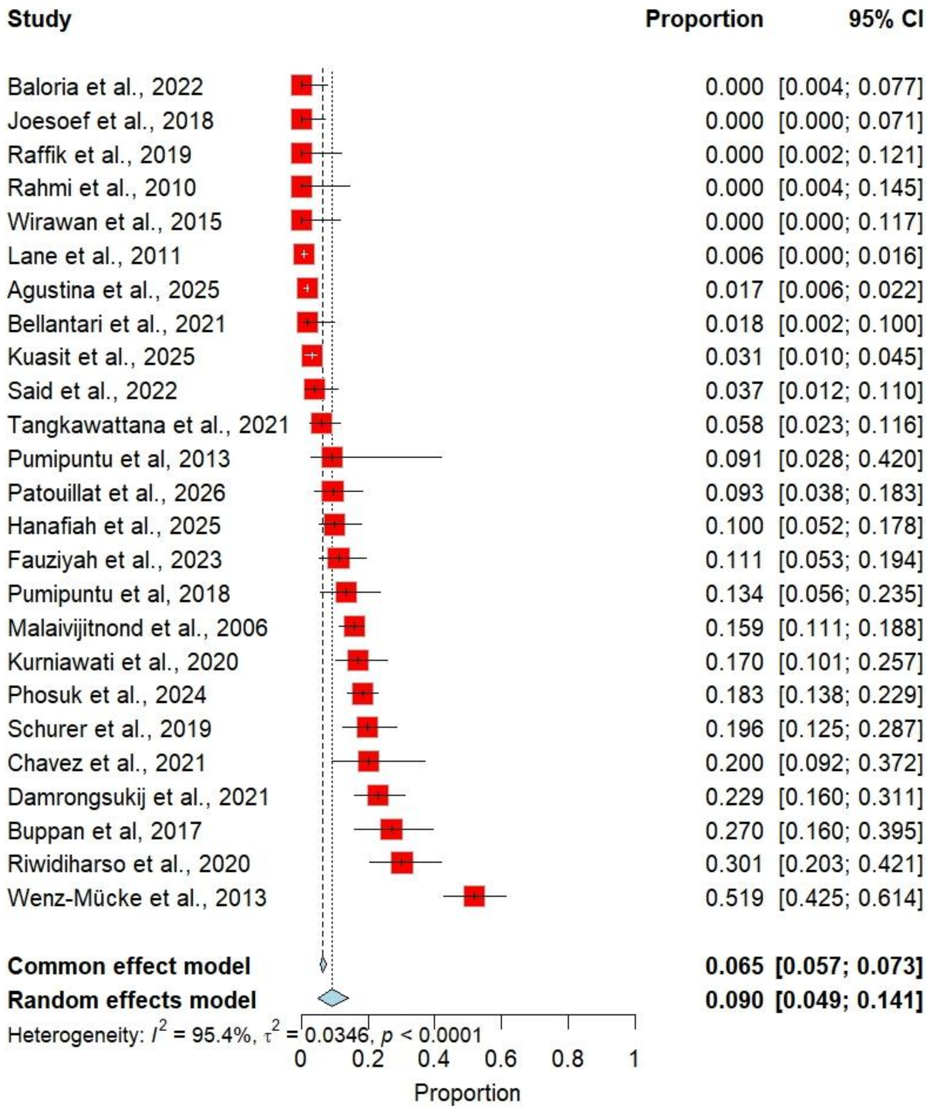

3. Sensitivity analysis including the excluded small-sample study for hookworm prevalence.

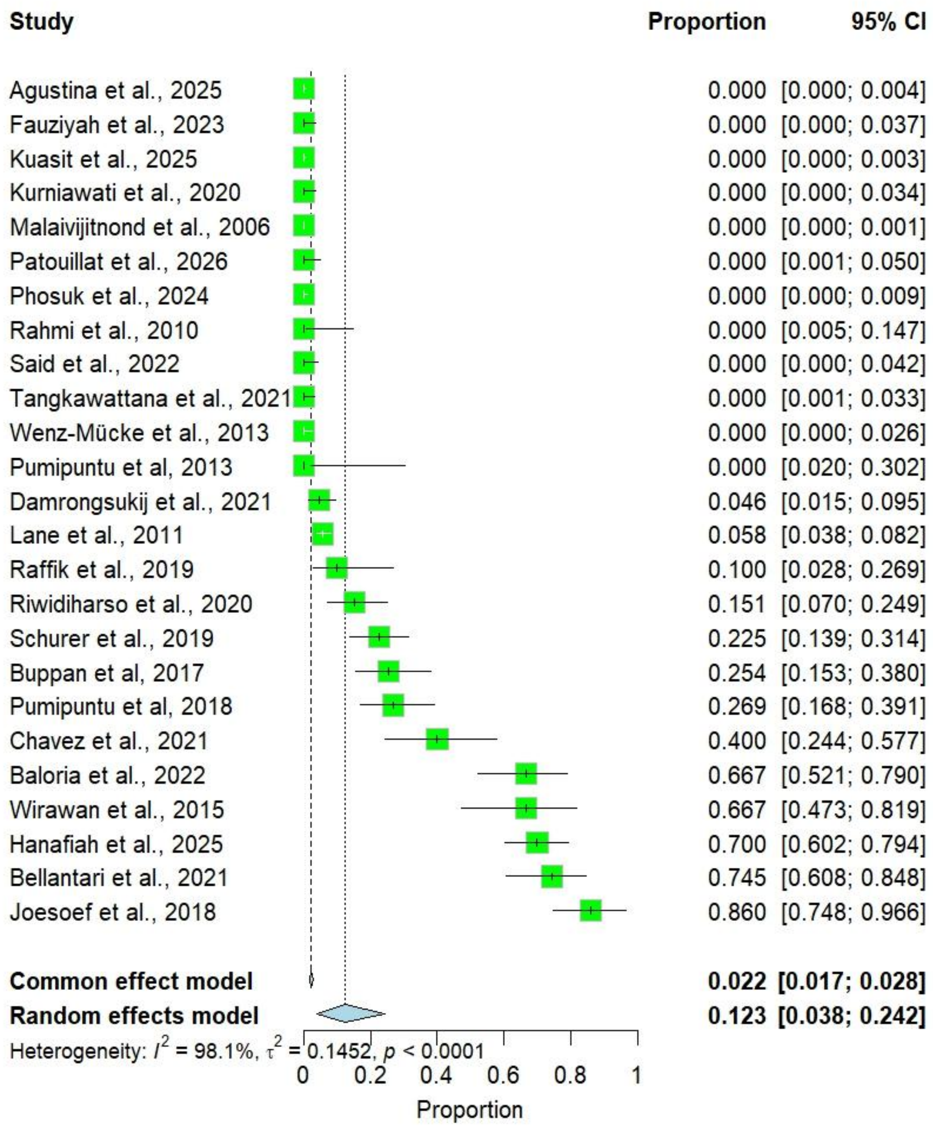

4. Sensitivity analysis including the excluded small-sample study for *Ascaris* spp. prevalence.

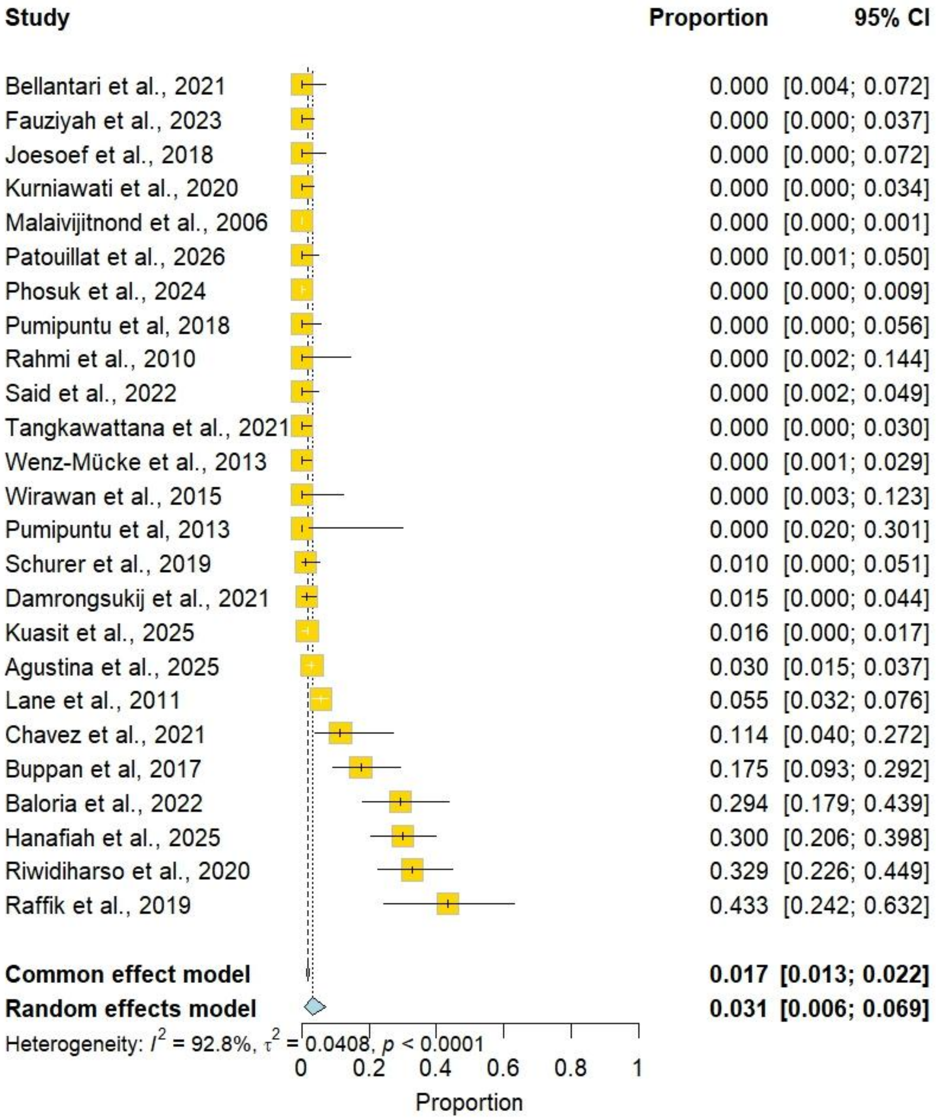

Supplement: Supplementary file 1 [file animals-16-01764-s001.zip › Supplementary file S4. Sensitivity analysis including the excluded small-sample study for STHs prevalence_edit.pdf]
