# Supplementary material for: Prevalence of Soil-Transmitted Helminths in Long-Tailed Macaques (Macaca fascicularis) in Asia: A Systematic Review and Meta-Analysis
Source: Animals (Basel). 2026 Jun 8;16(12):1764. doi: 10.3390/ani16121764 (PMC13295248; doi:10.3390/ani16121764)
Supplement: Supplementary file 1 [file animals-16-01764-s001.zip › Supplementary file S5. Funnel plots of STHs prevalence in free-ranging long-tailed macaque.pdf]

1. Funnel plots of hookworm prevalence in free-ranging long-tailed macaque populations

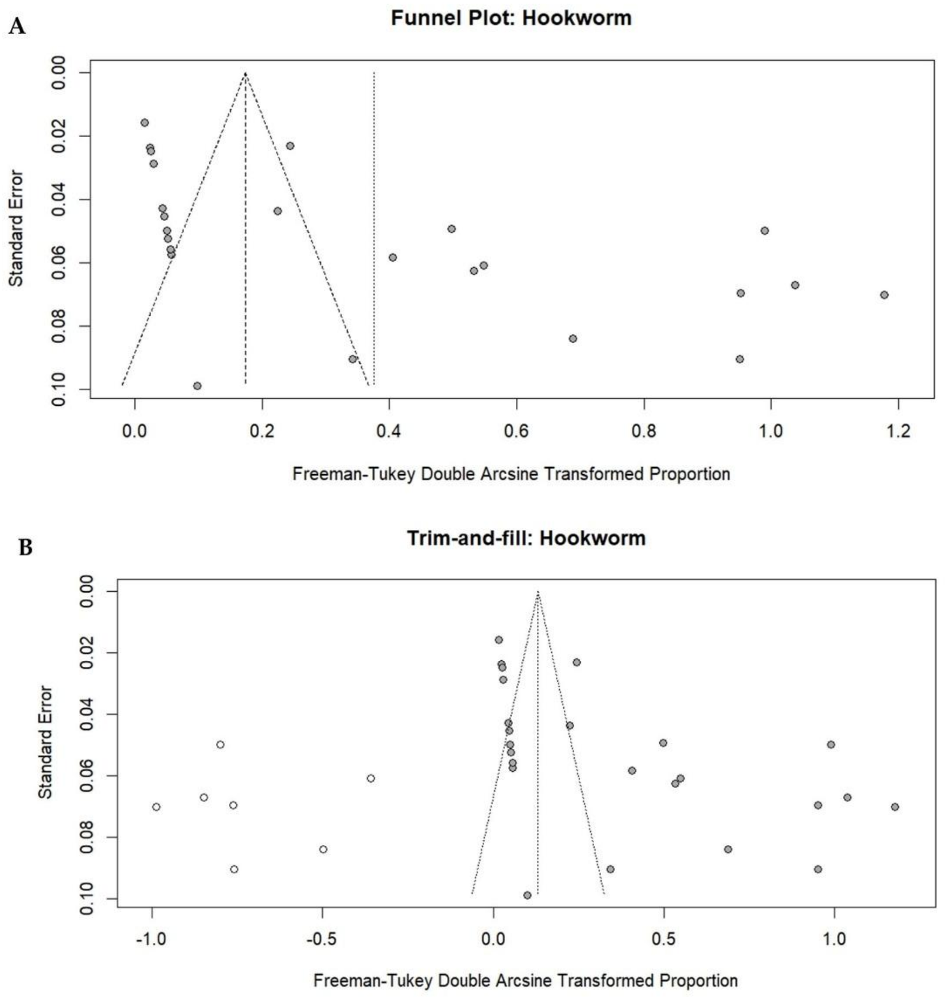

Test result: t = 3.99, df = 22, p-value = 0.0006, Bias estimate: 9.5849 (SE = 2.3999)

Number of studies: k = 31 (with 7 added studies)

	proportion	95%-CI
Random effects model	0.0104	[0.0000; 0.1032]

2. Funnel plots of *Strongyloides* spp. prevalence in free-ranging long-tailed macaque populations

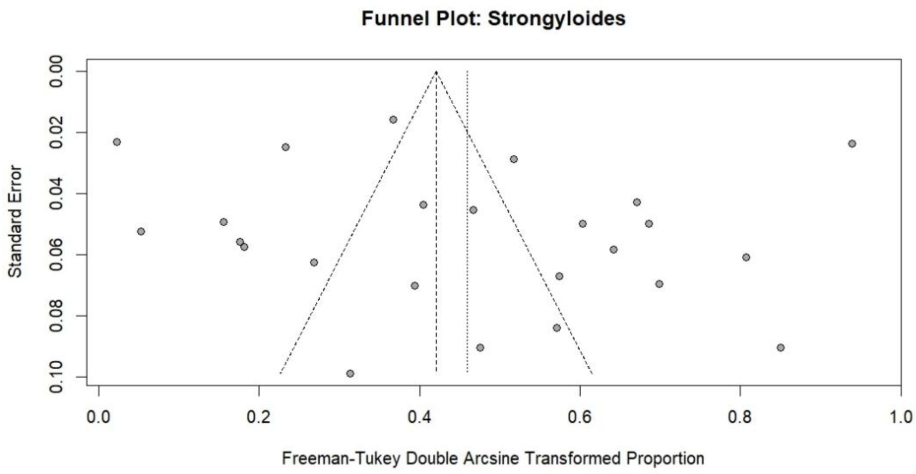

Test result: t = 0.66, df = 22, p-value = 0.5167, Bias estimate: 1.9736 (SE = 2.9946)

3. Funnel plots of *Trichuris* spp. prevalence in free-ranging long-tailed macaque populations

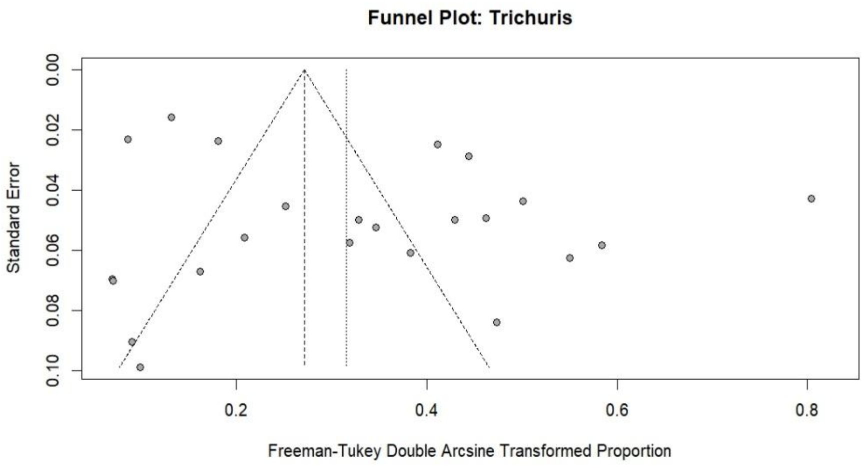

Test result: t = 1.67, df = 22, p-value = 0.1098, Bias estimate: 3.2007 (SE = 1.9206)

4. Funnel plots of *Ascaris* spp. prevalence in free-ranging long-tailed macaque populations

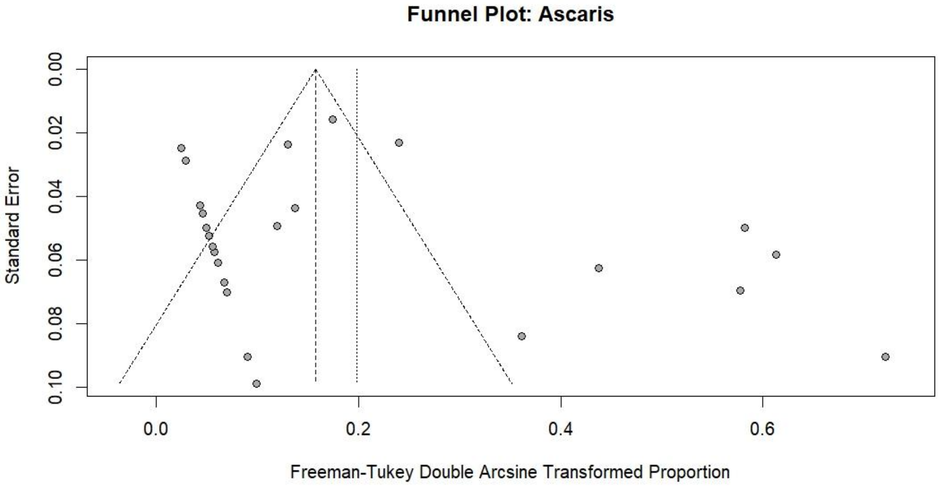

Test result: t = 1.05, df = 22, p-value = 0.3070, Bias estimate: 1.6580 (SE = 1.5855)
