# Supplementary material for: Prevalence of Soil-Transmitted Helminths in Long-Tailed Macaques (Macaca fascicularis) in Asia: A Systematic Review and Meta-Analysis
Source: Animals (Basel). 2026 Jun 8;16(12):1764. doi: 10.3390/ani16121764 (PMC13295248; doi:10.3390/ani16121764)
Supplement: Supplementary file 1 [file animals-16-01764-s001.zip › Supplementary file S6. Funnel plots of STHs prevalence in captive.pdf]

1. Funnel plots of *Trichuris* spp. prevalence in captive settings

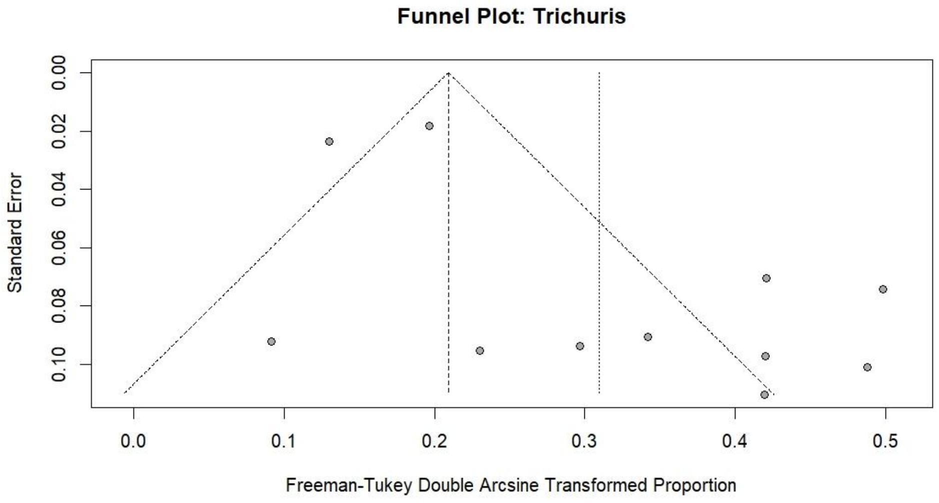

Test result: t = 2.95, df = 9, p-value = 0.0161, Bias estimate: 2.5036 (SE = 0.8473)

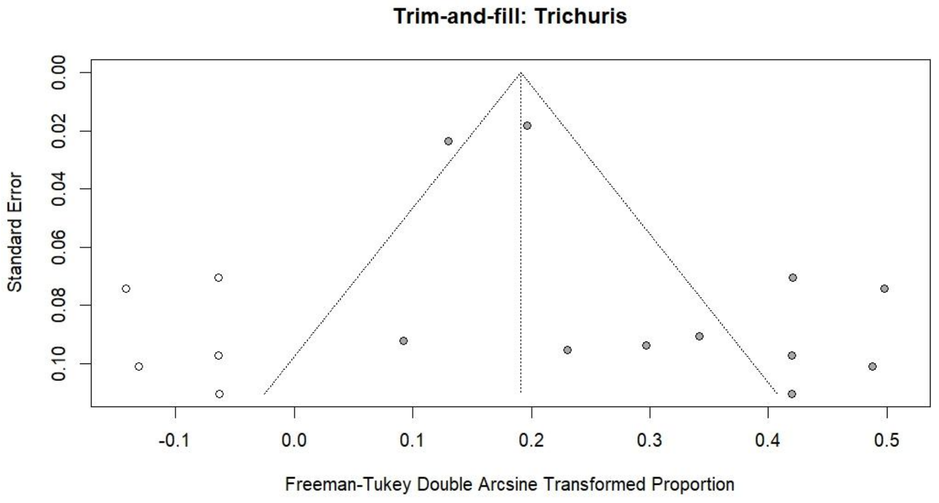

Trim-and-fill method to adjust for funnel plot asymmetry

Number of studies: k = 16 (with 5 added studies)

	proportion	95%-CI
Random effects model	0.0234	[0.0000; 0.0768]

2. Funnel plots of *Strongyloides* spp. prevalence in captive settings

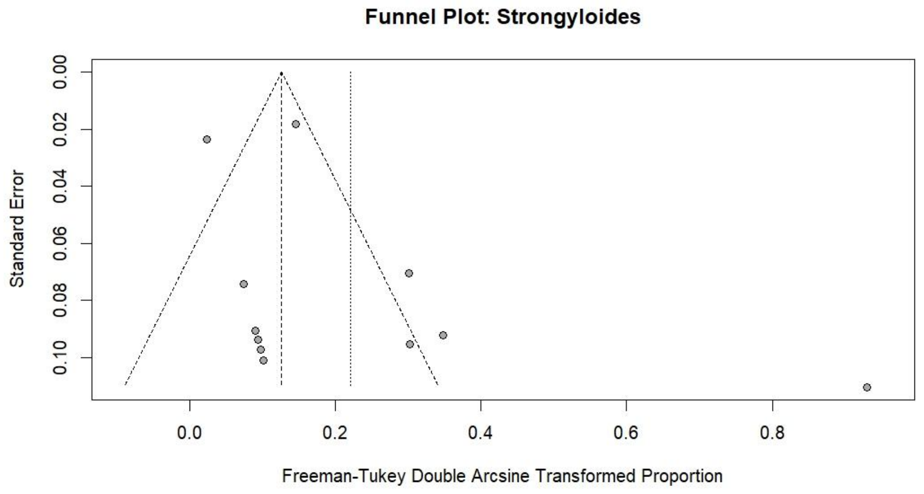

Test result: t = 1.49, df = 9, p-value = 0.1713, Bias estimate: 1.9911 (SE = 1.3394)

2. Funnel plots of hookworm prevalence in captive settings

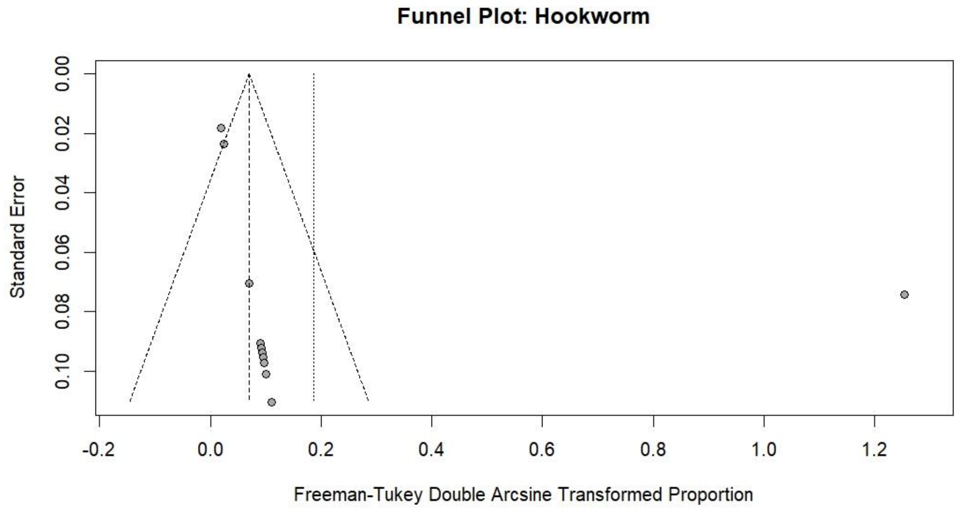

Test result: t = 1.24, df = 9, p-value = 0.2458, Bias estimate: 2.9703 (SE = 2.3926)

4. Funnel plots of *Ascaris* spp. prevalence in in captive settings

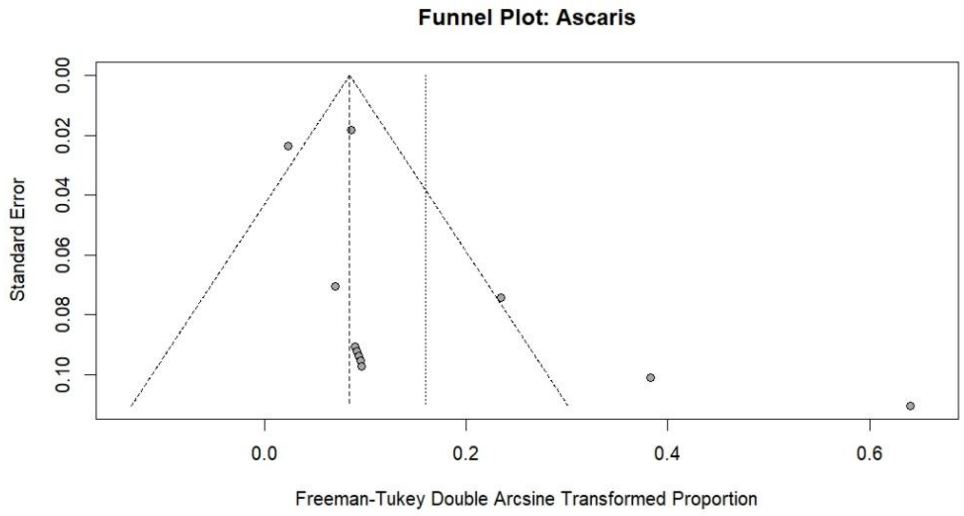

Test result: t = 1.99, df = 9, p-value = 0.0781, Bias estimate: 1.7564 (SE = 0.8838)
